# Integrating next-generation sequencing and artificial intelligence for the identification and validation of pathogenic variants in colorectal cancer

**DOI:** 10.3389/fonc.2025.1568205

**Published:** 2025-05-19

**Authors:** Juliana Rodriguez-Salamanca, Mariana Angulo-Aguado, Sarah Orjuela-Amarillo, Catalina Duque, Diana Carolina Sierra-Díaz, Nora Contreras Bravo, Carlos Figueroa, Carlos M. Restrepo, Andrés López-Cortés, Rodrigo Cabrera, Adrien Morel, Dora Janeth Fonseca-Mendoza

**Affiliations:** ^1^ School of Medicine and Health Sciences, Center for Research in Genetics and Genomics (CIGGUR), Institute of Translational Medicine (IMT), Universidad del Rosario, Bogotá, Colombia; ^2^ Coloproctology Department, Hospital Universitario Mayor - Méderi - Universidad del Rosario, Bogotá, Colombia; ^3^ Cancer Research Group (CRG), Faculty of Medicine, Universidad de Las Américas, Quito, Ecuador

**Keywords:** next generation sequencing (NGS), pathogenic germline variants, artificial intelligence, minigene assay, functional validation, colorectal cancer

## Abstract

**Background:**

Colorectal cancer (CRC) is recognized as a multifactorial disease, where both genetic and environmental factors play critical roles in its development and progression. The identification of pathogenic germline variants has proven to be a valuable tool for early diagnosis, the implementation of surveillance strategies, and the identification of individuals at increased cancer risk. Next-generation sequencing (NGS) has facilitated comprehensive multigene analysis in both hereditary and sporadic cases of CRC.

**Patients and methods:**

In this study, we analyzed 100 unselected Colombian patients with CRC to identify pathogenic (P) and likely pathogenic (LP) germline variants, classified according to the guidelines established by the American College of Medical Genetics and Genomics (ACMG) and the Association for Molecular Pathology (AMP). Using the BoostDM artificial intelligence method, we were able to identify oncodriver germline variants with potential implications for disease progression. We assessed the model’s accuracy in predicting germline variants by comparing its results with the AlphaMissense pathogenicity prediction model. Additionally, a minigene assay was employed for the functional validation of intronic mutations.

**Results:**

Our findings revealed that 12% of the patients carried pathogenic/likely pathogenic (P/LP) variants according to ACMG/AMP criteria. Using BoostDM, we identified oncodriver variants in 65% of the cases. These results highlight the significance of expanded multigene analysis and the integration of artificial intelligence in detecting germline variants associated with CRC. The average overall AUC values for the comparison between BoostDM and AlphaMissense were 0.788 for the entire BoostDM dataset and 0.803 for the genes within our panel, with individual gene AUC values ranging from 0.606 to 0.983. Functional validation through the minigene assay revealed the generation of aberrant transcripts, potentially linked to the molecular etiology of the disease.

**Conclusion:**

Our study provided valuable insights into the prevalence and frequency of P/LP germline variants in unselected Colombian CRC patients through NGS. Integrating advanced genomic analysis and artificial intelligence has proven instrumental in enhancing variant detection beyond conventional methods. Our functional validation results provide insights into the potential pathogenicity of intronic variants. These findings underscore the necessity of a multifaceted approach to unravel the complex genetic landscape of CRC.

## Introduction

Colorectal cancer (CRC) is the third most frequently diagnosed cancer worldwide and ranks as the second leading cause of cancer-related death (1, 2). Worldwide, approximately 1.9 million new CRC cases are reported, resulting in over 900,000 deaths globally (3, 4). Similarly, it has been projected that by the year 2024, approximately 2 million new cancer cases and 611.720 cancer-related deaths will occur in the United States, with an increase in the incidence of 6 of the 10 most common cancers. Notably, the incidence of CRC is estimated to rise by 1% to 2% annually among young adults (under 55 years of age) (5). About 1 in 23 men and 1 in 24 women are projected to be diagnosed with CRC at some point in their lives (6). While CRC is more common in developed countries, mortality rates have decreased due to early screening strategies such as colonoscopy and fecal occult blood tests (7). The incidence and mortality rates of colorectal cancer (CRC) in Colombia have been documented in several studies. According to Carvalho et al., there has been an increasing trend in CRC incidence in Cali, Colombia, with annual percentage increases of 2.8% for men and 3.3% for women between 1983 and 2012 (8). Regarding mortality, Piñeros et al. reported a rising burden of CRC-related deaths, with an estimated annual percentage change of 2% from 1984 to 2008 (9).

Additionally, according to GLOBOCAN, CRC accounts for approximately 11,163 new cases and 5,640 deaths annually in Colombia (3). In 2022, the country recorded 44,371 new cancer cases, of which 3,851 were attributed to CRC (10) (https://cuentadealtocosto.org/higia/).

Overall, these data highlight the increasing incidence and mortality rates of CRC in our country, underscoring the urgent need for improved preventive strategies and equitable access to healthcare services (11).

CRC is a complex disease in which genetic alterations and environmental risk factors play crucial roles in its development and progression (12, 13). Approximately 30% to 35% of patients with CRC report a family history of the disease, which can be attributed to genetic factors, common exposures, or both (14, 15). The familial component of CRC includes both hereditary syndromes and non-syndromic familial clustering, which can increase the risk of CRC even in absence of identifiable genetic mutations (16, 17). Only about 5% to 10% of CRC cases are due to high or moderate penetrance genetic variants associated with hereditary cancer syndromes such as Lynch syndrome *(MLH1*, *MSH2, MSH6, PMS2*, and *EPCAM*), familial adenomatous polyposis (*APC*), and MUTYH-associated polyposis (18). Patients carrying Pathogenic Variants (PVs) in these genes are subject to appropriate surveillance strategies, as recommended by the National Comprehensive Cancer Network (NCCN) guidelines for hereditary CRC (19).

Recent reports utilizing next-generation sequencing (NGS) to assess germline variants in multiple cancer-related genes have identified non-canonical pathogenic variants (PVs) potentially associated with CRC in both selected and unselected populations (18, 20, 21). Consequently, increasing the detection rate of PVs through NGS will enhance the identification of molecular alterations in CRC patients. In addition to genes associated with syndromes and genetic disorders conferring a high risk of CRC, other high-penetrance genes, such as *AKT, ATM, BMPR1A, BRAF, BRCA1/2, CHEK2, CTLA4, KRAS, MYO3A, PI3KCA, PTEN, RAS, SMAD2, SMAD3, TCF7L2, TGFBR2*, and *TP53*, as well as those classified as moderate- and lower-penetrance genes, should also be considered. This strategy can potentially improve the identification of risk conferred by PVs predisposing to CRC (22–24). The understanding of genetic mutations has been fundamental to the evolution of cancer therapies, enabling the development of targeted treatments that inhibit key oncogenic proteins and driving the creation of antibody-drug conjugates that combine monoclonal antibodies with cytotoxic agents. In addition, it has contributed to the advancement of immunotherapy and cellular therapies such as CAR-T cells. These personalized strategies have improved treatment efficacy and reduced side effects, representing a milestone in modern oncology (25).

In Colombia, studies focused on germline variants in CRC have primarily concentrated on syndromic genes such as *APC*, *MLH1*, and *TP53* (26–29). However, the lack of comprehensive genomic data and information on population frequency makes it challenging to construct a genomic profile for CRC predisposition in the Colombian population. The aim of this research was to identify molecular variants potentially related to the disease in Colombian patients with unselected CRC and to determine their frequency in CRC-associated genes. The study analyzed 100 CRC-affected patients, generating a virtual gene panel evaluated through NGS, which included 206 genes of interest. Additionally, intronic variants were validated using functional minigene assays.

## Materials and Methods

### Sampling and Data collection

This study included 100 unselected patients, defined as individuals diagnosed with cancer who have not been pre-screened or stratified based on specific clinical, molecular, or demographic characteristics. All patients had a histopathological diagnosis of colorectal cancer (CRC) and received care at the Coloproctology Service of Méderi Hospital in Bogotá, Colombia. Patients over 18 years of age with a confirmed biopsy of any type of CRC were eligible, invited to participate in the study, and provided informed consent. Sociodemographic and clinical data were collected through interviews and review of clinical records. The variables examined included sex, age, comorbidities (hypertension, diabetes mellitus, chronic obstructive pulmonary disease, cancer, and others), family history of cancer, lifestyle habits, CRC screening tests, height, weight, age at diagnosis, tumor location, lymphovascular infiltration, tumor stage, and metastasis. The sample size was convenience-based, including patients treated at Méderi Hospital during a defined period from 2020 to 2022. All experimental procedures were approved by the Ethics Committee of Universidad del Rosario and were conducted according to the principles outlined in the Declaration of Helsinki (DVO005 1607-CV1436).

### NGS - Whole-Exome sequencing

The patient´s DNA was extracted, from peripheral blood, using the Quick-DNA 96 plus kit (Zymo Research). The quality and quantity of DNA were determined using the Quantifluor ONE dsDNA system on a GloMax Discover instrument (Promega). Library preparation was carried out with 250 ng of DNA using the MGIEasy FS DNA Library Prep Kit. Enzymatic DNA fragmentation was performed to obtain fragments ranging from 200 to 400 bp, followed by end repair and PCR amplification. Specific regions of interest were captured using the Exome Capture V5 probe and streptavidin beads. Specific primers were employed for enrichment in the final PCR reaction. For sequencing, the DNA was circularized, and the library was denatured after split oligo ligation, followed by digestion and purification using specific beads. The circularized DNA was used to generate DNBs (nanoballs) through the rolling circle amplification process (https://en.mgi-tech.com/). DNBs were quantified and subsequently sequenced on the DNBSeqG400 platform. The obtained reads were mapped to the hg19 reference genome using the Burrows-Wheeler Aligner (BWA) and organized using SAMtools (https://github.com/samtools/samtools). Duplicate reads were identified and removed using Picard Software (https://broadinstitute.github.io/picard/). Coverage and depth analysis were carried out using BAMBA tool, we considered 50X as an acceptable threshold. >93% of total bases called had a Phred-scaled quality score greater than 30 (>Q30).

A minimum of 7 Gb raw data was obtained and the percentage of reads properly mapped was >99.99% (56,446,362-101,662,468) per sample. Average mapping efficiency was >99%, with sequencing depth on target and coverage of target region >50x and >94%, respectively. The average fraction of target covered with >20x was >72%. Coverage uniformity (10x) was ≥90% and the average fraction of target covered with at least 10x, 20x, 50x and 100x was >90%, >72%, >40% and >19%, respectively. The average number of paired ends reads that mapped to the reference genome was 72,351,272 (99.99%).

The library preparation and sequencing were performed by GencellPharma (Bogota, Colombia). The analyzed panel included 206 genes selected based on evidence from case-control association studies, systematic reviews, GWAS and functional validation studies in CRC, considering their biological relevance, the implication in physio pathological processes, and their roles in oncogenesis (this group was considered as candidate genes). Additionally, genes included in diagnostic panels from CGC genetics, ICM Atrys division, Mayo Clinic Laboratories, GENDIA-genetics and molecular biology, CD Genomics disease panel, Centogene, and Invitae were incorporated. The final panel consisted of 102 genes from established diagnostic panels and 104 candidate genes (**Table 1**).

**Table 1:** Extended multigene panel (n=206 genes).

Molecular diagnosis panel				Gene-candidate panel			
*AIP*	*EGFR*	*NF2*	*RUNX1*	*ACTR1B*	*EDN1*	*MAMSTR*	*SBF2*
*ALK*	*EPCAM*	*NOTCH2*	*SDHA*	*ALCAM*	*EIF3H*	*MGMT*	*SEMA4A*
*APC*	*EPM2A*	*NOTCH3*	*SDHAF2*	*APE1*	*ERCC1*	*MRE11*	*SF3A3*
*ATM*	*FAN1*	*NRAS*	*SDHB*	*ARFGEF2*	*EXO1*	*MRE11A*	*SFMBT1*
*AXIN2*	*FH*	*NTHL1*	*SDHC*	*ATF1*	*FAM109A*	*MYC*	*SH2B3*
*BAP1*	*FLCN*	*PALB2*	*SDHD*	*ATXN2*	*FANCC*	*MYO3A*	*SHROOM2*
*BARD1*	*GALNT12*	*PDGFRA*	*SMAD4*	*B9D2*	*FANCE*	*NABP1*	*SLC15A4*
*BLM*	*GATA2*	*PHOX2B*	*SMARCA4*	*BMP2*	*FEN1*	*NCAPG*	*SLC6A18*
*BMPR1A*	*GPC3*	*PIF1*	*SMARCB1*	*BMP4*	*FKBP5*	*NXN*	*SMAD6*
*BRAF*	*GREM1*	*PIK3CA*	*SMARCE1*	*BMP5*	*FMN1*	*OGG1*	*SMAD7*
*BRCA1*	*HOXB13*	*PMS1*	*STK11*	*BORA*	*FUT2*	*PIAS1*	*SMAD9*
*BRCA2*	*HRAS*	*PMS2*	*SUFU*	*C11orf53*	*GLI3*	*PITX1*	*SMARCD1*
*BRIP1*	*KIT*	*POLD1*	*TELO2*	*CABLES2*	*GNL1*	*PLCB1*	*TBX3*
*CASR*	*KRAS*	*POLE*	*TERC*	*CCND2*	*HHIP*	*PLGLA*	*TCF7L2*
*CCND1*	*MAX*	*POT1*	*TERT*	*CD44*	*HNF4A*	*PNKD*	*TFEB*
*CDC73*	*MEN1*	*PRKAR1A*	*TGFBR2*	*CDKN2B*	*IL12RB1*	*POLD3*	*TLE4*
*CDH1*	*MET*	*PTCH1*	*TMEM127*	*CHRDL2*	*KDR*	*POU5F1B*	*TMBIM1*
*CDK4*	*MITF*	*PTEN*	*TP53*	*COL4A2*	*KLF5*	*PRDM1*	*TMEM59*
*CDKN1B*	*MLH1*	*RAD50*	*TSC1*	*COLCA1*	*LAMA5*	*PREX1*	*TNS3*
*CDKN1C*	*MLH3*	*RAD51C*	*TSC2*	*COLCA2*	*LAMC1*	*PTPN1*	*TOX2*
*CDKN2A*	*MSH2*	*RAD51D*	*VHL*	*COX14*	*LGR5*	*PTPN12*	*TP53BP1*
*CEBPA*	*MSH3*	*RB1*	*WRN*	*CRTC3*	*LIG1*	*RBBP8*	*TTC22*
*CHEK2*	*MSH6*	*RBL1*	*WT1*	*CTNNB1*	*LIMA1*	*RHPN2*	*VTI1A*
*CTNNA1*	*MUTYH*	*RECQL4*	*XAF1*	*DCLRE1C*	*LIMK2*	*RPS20*	*WNT4*
*DICER1*	*NBN*	*RET*	*DIP2B*	*LRP1*	*RTEL1*	*XRCC2*
*DIS3L2*	*NF1*	*RNF43*	*DUSP10*	*MACC1*	*SATB2-AS1*	*ZAP70*

### Bioinformatic Analysis

Variant call format (VCF) files were analyzed using the software VarSeq^®^ (Golden Helix, v 2.3.0). We incorporated the following database annotations: ClinVar (https://www.ncbi.nlm.nih.gov/clinvar/), Ensembl (https://www.ensembl.org/index.html), RefSeq (https://www.ncbi.nlm.nih.gov/refseq/), dbNSFP Functional predictions, dbSNP, REVEL, OMIM Phenotype Ontology (https://www.omim.org/), UniProt Variants (https://www.uniprot.org/) and gnomAD v2.1 (https://gnomad.broadinstitute.org/).

**Figure 1:**
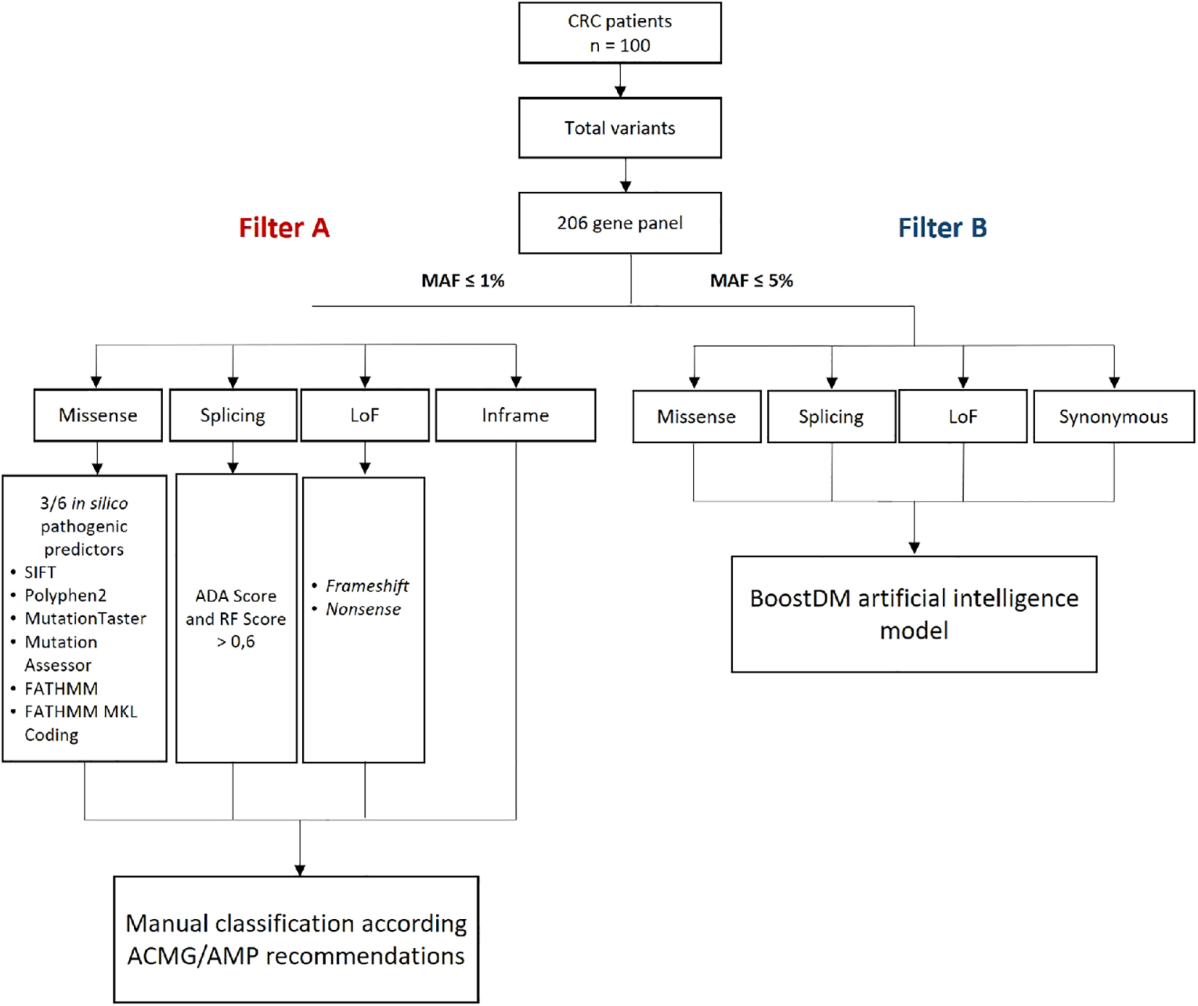
Methodological scheme for filtering genomic variants in CRC patients. ADA, adaptative boosting; CRC, colorectal cancer; MAF, minor allele frequency; n, number of patients; LoF, loss of function; RF, random forest. ACMG/AMP, American College of Medical Genetics and Genomics and the Association for Molecular Pathology.

To identify pathogenic/likely pathogenic (P/LP) and oncodriver variants in the 206 CRC-related genes, we applied a bioinformatic analysis using two filtering and classification strategies: (A) manual classification according to the ACMG/AMP recommendations (30, 31), and (B) the BoostDM artificial intelligence system (32) (**Figure 1**). For strategy A, we prioritized molecular variants with a minor allele frequency (MAF) of ≤1%, including loss-of-function (LoF) variants (nonsense, frameshift, and splice site), in-frame, and missense mutations. The potential functional impact of splicing variants was assessed using scores from the adaptive boosting (ADA) and random forest (RF) algorithms (cut-off ≥ 0.6). For missense variants, we prioritized those with positive *in silico* pathogenicity predictions in at least 3 out of 6 predictors integrated into the VarSeq^®^ software (SIFT, Polyphen2, Mutation Taster, Mutation Assessor, FATHMM, and FATHMM MKL Coding). Variants classified as functionally relevant were subsequently manually classified following the ACMG/AMP recommendations. The final dataset included variants that met the criteria for P and LP as defined by Hampel et al. (30). For strategy B, all variants with a MAF of less than 5% were analyzed using the BoostDM artificial intelligence model. The final dataset included variants classified as oncodriver mutations, as described by Muiños et al. (32). Furthermore, we sought to evaluate its potential in predicting the significance of germline variants in colorectal cancer genes. For benchmarking against a high-performing germline variant classifier, we utilized AlphaMissense (33), which demonstrates leading-edge performance in predicting the pathogenicity of missense variants, by integrating structural context and evolutionary conservation. This tool is validated by extensive genetic and experimental benchmarks and can classify a vast majority of missense variants with a high precision score on recognized databases like ClinVar, without being explicitly trained on such data (33). A Python script (S1 Appendix) was developed to assign AlphaMissense classifications (Obtained from https://storage.googleapis.com/dm_alphamissense/AlphaMissense_hg38.tsv.gz) to all variants scored for BoostDM available in the public repository (https://www.intogen.org/boostdm/downloads). Utilizing this script, precision-recall curves were generated, and AUC scores were calculated to evaluate the performance of BoostDM’s predictions of variants classified by AlphaMissense. This comparison utilized saturation mutagenesis data, encompassing 81 genes, including 22 that are part of our colorectal cancer gene panel. Genes without AlphaMissense predictions were excluded.

### Population Genetic Analysis

For each variant identified through NGS analysis, we assessed the allelic frequency, genotypic frequency, and the Hardy Weinberg equilibrium (HWE) using the SNP-Stats software (https://www.snpstats.net/start.htm). Deviation from HWE was determined using χ2 goodness-of-fit test with 1° of freedom. The allele frequencies of the study were compared to the global and Latin-American populations, obtained from the gnomAD database (https://gnomad.broadinstitute.org), and statistical significance was assessed using chi-square (χ2) test, with significance being established at *p-value <*0.05.

### Plasmid constructs

We used patient genomic DNA to amplify the region encompassing the exon nearest to the mutation, along with 300 bp of flanking intronic sequences (upstream and downstream of the exon). Primers were designed according to Putscher et al., 2021 and verified by Primer-BLAST (34). The PCR was performed using the New England Biolabs Q5 master mix (Q5^®^ High-Fidelity 2X Master Mix cat: M049), according to the manufacturer´s protocol. The PCR products were recombined with the pSpliceExpress vector using Gateway™ BP Clonase™ II Enzyme mix (Invitrogen), following the manufacturer’s instructions. The sequence of the constructed vector was confirmed through Sanger sequencing.

### Cell culture transfection

HCT-116 and HEK-293 cell lines, were cultured in Dulbecco’s Modified Eagle’s medium (DMEM) supplemented with 10% fetal bovine serum and 5% penicillin/streptomycin at 37°C in a 5% CO_2_ environment. Both cell lines were seeded at a density of 60,000 cells per well into a 24 well-plate, with triplicates for each experimental condition. 1 µg of the vector was transfected into the cells and incubated for 48 hours using FuGENE^®^ 6 Transfection Reagent (Promega), according to the manufacturer’s protocol.

### Total RNA extraction and RT-PCR analysis

Total RNA was extracted using the TRIzol^®^ Reagent protocol. 500 ng of RNA was used to synthesize cDNA with SuperScript III cDNA first strand (Invitrogen), previous treatment with DNase I Amplification Grade (Sigma-Aldrich). Using primers targeting exons 2 and 3 of rat insulin (whose sequences are located into the pSpliceExpress vector and surrounds the sequences cloned through recombination with Gateway™ BP Clonase™ II Enzyme mix). PCR was performed under the following conditions: 95°C for 1 minute, 60°C for 40 seconds, and then 3 minutes at 72°C for 30 cycles. RT-PCR products were visualized on 1.5% agarose gels. The obtained bands were analyzed using densitometry with Image Lab software (BIO-RAD). For each band, obtained in the Wild-Type (WT) and mutant (MUT) RT-PCR assays, intensity measurement was performed, allowing subsequent establishment of differences in the transcripts obtained.

Additionally, the RT-PCR products for both WT and MUT were cloned in the storage vector, pCR4-TOPO, using the TOPO^®^ TA Cloning Kit for Sequencing (Invitrogen) following the manufacturer’s instructions. After transformation in the One Shot Top 10 chemically competent cells (https://www.thermofisher.com/), 10 colonies were randomly selected for Sanger sequencing following plasmid DNA extraction.

### Statistical Analysis

We determined the association between non-genetic factors and being a carrier of a Pathogenic, Likely Pathogenic and oncodriver variant using a bivariate analysis. Statistical significance was determined at a threshold of *p-value <*0.05.

To compare WT vs MUT for experimental assays, an independent samples t-test (Student’s t-test) was applied for comparisons between the two experimental groups and their replicates, using SPSS V.29 and GraphPad Prism 10 software. A statistically significant difference was considered if *p-value* < 0.05.

## Results

### Demographic and clinicopathological characteristics

This study included 100 unselected patients with a histopathological diagnosis of CRC, and the characteristics of the population are summarized in **Table 2**. Women constituted the majority, representing 55% (n=55) of the patients. A family history of cancer was reported in 63% of individuals, with 11% of cases involving CRC. Regarding tumor location, there was a higher prevalence of right-sided colon cancer, followed by sigmoid and rectal cancer (44% and 29%, respectively). The sample analyzed exhibited heterogeneous histology and tumor staging, with a greater proportion of moderately differentiated adenocarcinoma and stage II tumors (30% and 36%, respectively). Most patients did not present metastasis at the time of inclusion in the study (76%). The mean age at CRC diagnosis was 65.5 years, with 12% of patients diagnosed before the age of 50. An association analysis of clinical variables grouped by age at diagnosis revealed statistically significant differences in family history of CRC (p<0.05) (**Table 2**).

**Table 2:** Demographic and clinicopathological characterization.

Characteristics	Total	Age at diagnosis		P-value	P-value Yates correction
		< 50	≥ 50		
No. of patients	100	12	88		
Gender				0.035*	0.073
Male	45	2	43		
Female	55	10	45		
Age at CRC diagnosis					
Mean (SD)	65,5 (12,6)	41,25 (8,4)	68,8 (8,9)		
**Family history of cancer**				0.632	0.889
No	32	3	29		
Yes	63	8	55		
Unknown	5	1	4		
**Family history of CRC**				0.004*	0.017*
No	81	6	75		
Yes	11	4	7		
Unknown	8	2	6		
Primary tumor site					
Right sided colon cancer	44	2	42	0.042*	0.085
Sigmoid	29	6	23	0.087	0.171
Rectum	17	4	13	0.108	0.232
Entire colon	1	0	1	0.711	1
Multiple primary colorectal cancer	9	0	9	0.246	0.533
Tumor histological stage					
Well differentiated adenocarcinoma	7	0	7	0.311	0.682
Moderately differentiated adenocarcinoma	30	4	26	0.788	1
Poorly differentiated adenocarcinomald	1	0	1	0.711	1
Mucinous adenocarcinoma	3	1	2	0.248	0.801
Infiltrating adenocarcinoma	3	1	2	0.248	0.801
Multiple histological stage	56	6	50	0.655	0.892
**Cancer Stage**				0.484	0.484
0	2	1	1		
I	14	1	13		
I	36	4	32		
III	32	3	29		
IV	15	2	13		
Unknown	1	1	0		
**Lymphovascular infiltration**				0.188	0.319
Yes	53	8	45		
No	45	3	42		
Unknown	2	1	1		
**Metastasis**				0.719	1
Yes	22	2	20		
No	76	9	67		
Unknown	2	1	1		
**Pathogenic or Likely Pathogenic Variant (ACMG/AMP)**				0.140	0.315
Yes	12	3	9		
No	88	9	79		
**Oncodriver Variant (BoostDM)**				0.156	0.273
Yes	65	10	55		
No	35	2	33		

*Statistical significance.

### Germline mutation landscape of Colombian CRC patients

#### Molecular variants identified following ACMG/AMP recommendations

**Figure 2:**
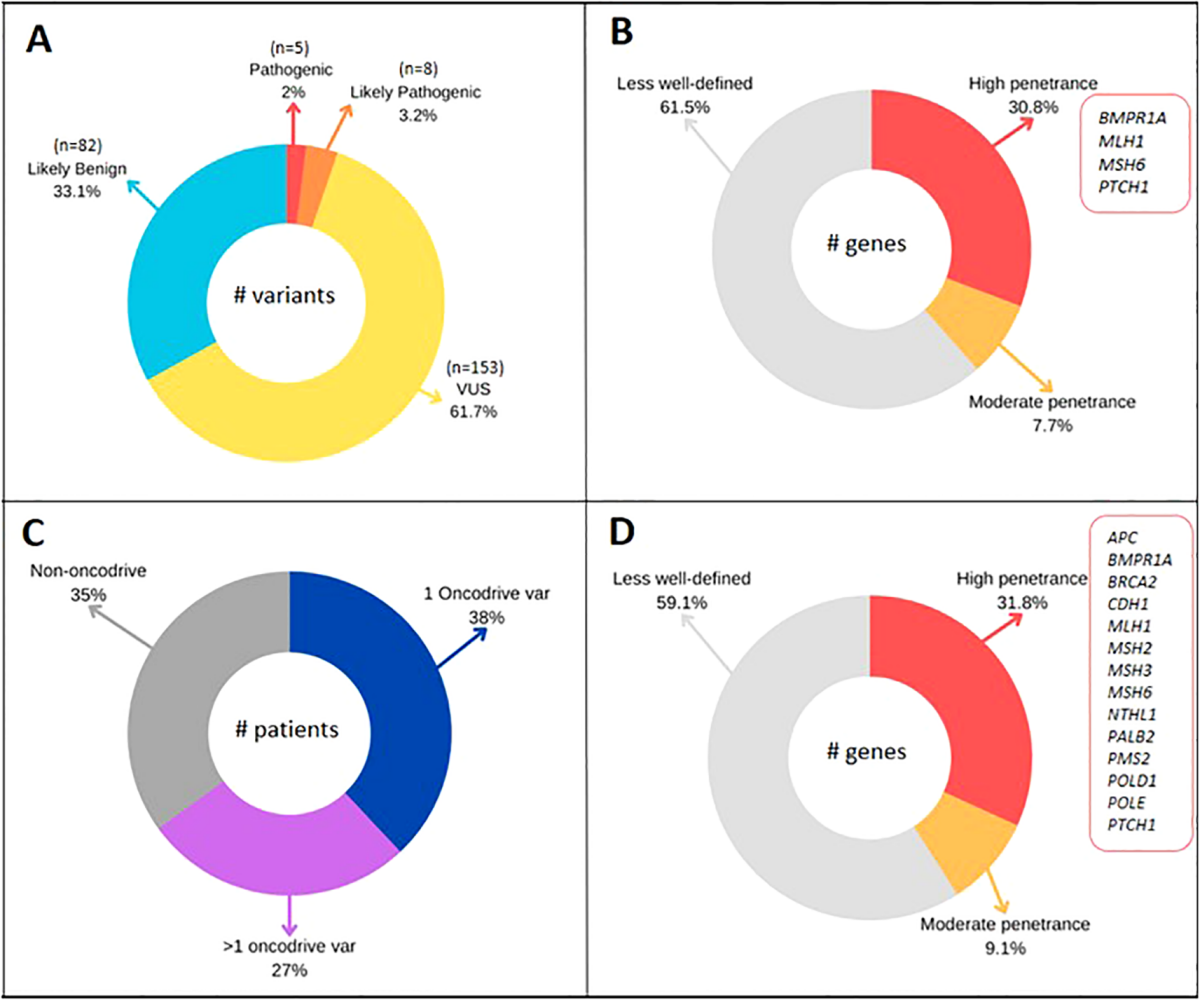
**(A)** Variants classified by ACMG/AMP classification in 100 CRC patients. **(B)** Penetrance genes of P/LP variants (Filter A). **(C)** Patients with 1 or >1 oncodriver variant classified with BoostDM model. **(D)** Penetrance genes of oncodriver variants (Filter B).

Following the bioinformatics pipeline described in the methodology (**Figure 1**) and applying Filter A, which identified pathogenic (P) and likely pathogenic (LP) variants according to ACMG/AMP recommendations, our study detected a total of 248 variants (**Figure 2A**). Of these, 5 (2%) were classified as pathogenic (P), 8 (3.2%) as likely pathogenic (LP), 153 (61.7%) as variants of uncertain significance (VUS), and 82 (33.1%) as likely benign (LB) (**Table 3** and **Figure 2A**). The detection rate for P/LP variants was 12% (12 patients). A total of 11% of patients carried a single P/LP mutation in a heterozygous state, while one patient was identified with two variants, *MLH1* c.1039delA and *IL12RB1* c.1791 + 2T>G, classified as LP and P, respectively.

**Table 3:** Described pathogenic and likely pathogenic germline variants in CRC patients – Filter A (ACMG/AMP).

Penetrance	Gene panel	Gene	Transcript	Variant	Protein	rs ID	Zygosity	Type	ACMG/AMP classification	Criteria	Count
High	Diagnosis	*BMPR1A*	NM_004329.3	c.176T>A	p.Leu59Ter	rs1564714834	Het	Missense	Pathogenic	PVS1 + PM2 + PM4 + BP1	1
High	Diagnosis	*MLH1*	NM_000249.4	c.1039delA	p.Thr347Leufs*20	Not reported	Het	LOF	Likely Pathogenic	PVS1 + PM2	1
High	Diagnosis	*MSH6*	NM_000179.3	c.3516_3517delAG	p.Arg1172Serfs*4	rs398123232	Het	LOF	Pathogenic	PVS1 + PM2 + PM4	1
High	Diagnosis	*PTCH1*	NM_000264.5	c.3241G>A	p.Val1081Met	rs587778629	Het	Missense	Likely Pathogenic	PS3 + PM2 + PP3	1
Less well-defined	Diagnosis	*FLCN*	NM_144997.7	c.1285delC	p.His429Thrfs*39	rs80338682	Het	LOF	Likely Pathogenic	PVS1 + PM2	1
Less well-defined	Diagnosis	*NOTCH3*	NM_000435.3	c.1345C>T	p.Arg449Cys	rs762734007	Het	Missense	Likely Pathogenic	PM1 + PM2 + PP2 + PP3	1
Less well-defined	Diagnosis	*NTHL1*	NM_002528.7	c.244C>T	p.Gln82Ter	rs150766139	Het	LOF	Pathogenic	PVS1 + PM2 + PM4	1
Moderate	Diagnosis	*BARD1*	NM_000465.4	c.2229dupT	p.Asn744Ter	rs1259296823	Het	LOF	Pathogenic	PVS1 + PM1 + PM2 + PM4	1
Less well-defined	Candidate	*ERCC1*	NM_202001.3	c.702+1G>A	–	rs747911302	Het	Splice	Likely Pathogenic	PVS1 + PM2	1
Less well-defined	Candidate	*EXO1*	NM_130398.4	c.1465delA	p.Arg489Glyfs*32	Not reported	Het	LOF	Likely Pathogenic	PVS1 + PM2	1
Less well-defined	Candidate	*IL12RB1*	NM_005535.3	c.1791+2T>G	–	rs554063682	Het	Splice	Pathogenic	PVS1 + PM2 + PP3	1
Less well-defined	Candidate	*OGG1*	NM_002542	c.137G>A	p.Arg46Gln	rs104893751	Het	Missense	Likely Pathogenic	PS3 + PM2	1
Less well-defined	Candidate	*SMAD9*	NM_001127217.3	c.781+2T>A	–	rs770716081	Het	Splice	Likely Pathogenic	PVS1 + PM2 + BP4	1

Het, Heterozygous; LOF, Loss of function.

Among the identified P/LP variants in the studied population, 38.5% (5/13) were in genes not typically included in routinely used diagnostic panels in clinical practice. These variants corresponded to *ERCC1:* c.702 + 1G>A, *EXO1:* c.1465delA, *IL12RB1:* c.1791 + 2T>G, *OGG1:* c.137G>A, and *SMAD9:* c.781 + 2T>A (**Table 3**). Of the P/LP variants, 61.5% (8/13) were found in genes commonly selected in molecular diagnostic panels for cancer. Four of these were in high-penetrance genes *(BMPR1A:* c.176T>A, *MLH1:* c.1039delA, *MSH6:* c.3516_3517delAG, and *PTCH1:* c.3241G>A), while one was in the moderate-penetrance gene *BARD1:* c.2229dupT. For eight of the analyzed genes where variants of interest were found, the penetrance has not been clearly defined (*FLCN, NOTCH3, NTHL1, ERCC1, EXO1, IL12RB1, OGG1*, and *SMAD9*) (**Table 3** and **Figure 2B**).

Regarding variant types, we observed six loss-of-function (LoF) variants, four missense variants, and three splice-site variants. Notably, two LP variants were novel and had not been previously reported in public databases such as gnomAD (*MLH1*: c.1039delA, p. Thr347Leufs*20, and EXO1: c.1465delA, p. Arg489Glyfs*32) (**Table 3**). All variants classified as P/LP were confirmed through Sanger sequencing.

#### Molecular Variants identified for BoostDM model

**Figure 3:**
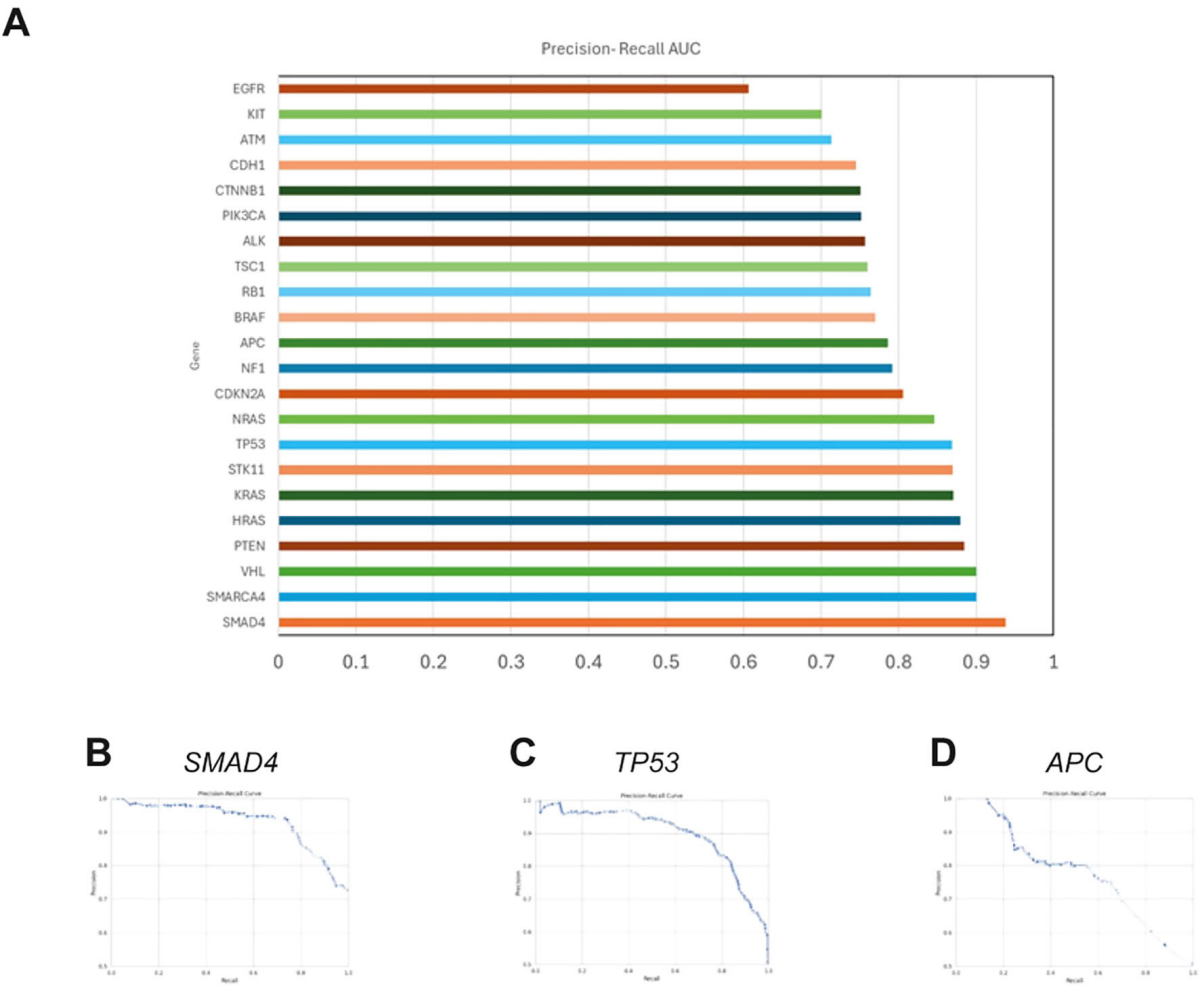
Comparative analysis of BoostDM and AlphaMissense predictions **(A)**. Bar chart displaying the average area under the precision-recall curve (AUC) for each gene analyzed by BoostDM, reflecting the model’s ability to distinguish between benign and non-benign variants as classified by AlphaMissense. When there are BoostDM analyses for multiple cancer types for variants within a gene, the AUC represents the average value across tumor types. **(B–D)**. Representative precision-recall curves for colorectal adenocarcinoma (COREAD) BoostDM predictions of AlphaMissense classifications for *TP53*, *SMAD4* and *APC*, showing AUCs of 0.931, 0.898, and 0.777 respectively. Similar results were obtained for all genes and cancer contexts evaluated.

The BoostDM model, developed by Muiños et al. in 2021, is a machine learning-based methodology designed for evaluating the oncogenic potential of mutations. We tested whether this model can accurately predict germline variants by evaluating its agreement with the AlphaMissense pathogenicity prediction model, which has shown state-of-the-art performance for predicting germline disease variants (33). We observed average overall AUC values for the comparison between BoostDM and AlphaMissense of 0.788 for the entire BoostDM dataset and 0.803 for the genes within our panel, with AUC values for individual genes ranging from 0.606 to 0.983 (**Figure 3**) using publicly available saturation mutagenesis data (S1 Appendix). The reliability of BoostDM in our study underscores its potential applicability beyond somatic mutations, suggesting it may serve as an informative tool for germline variant analysis in CRC.

Applying this model facilitated the identification of 68 oncodriver variants across 43 genes (**Supplementary Table 1**), which were found in 65 of the analyzed patients, resulting in a detection rate of 65%. Notably, 27% of individuals were found to carry more than one mutation (**Figure 2C**). Of the variants identified through this analysis, 72% were associated with genes included in cancer diagnostic panels, while the remaining 28% were linked to candidate genes. Additionally, 18.3% (8/43) of the genes with oncodriver variants are related to syndromic CRC, and 20.5% (14/68) of the study’s variants were identified in these genes. Penetrance has been clearly defined for 18 of the genes in which oncodriver mutations were identified, with 14 being high-penetrance and 4 moderate-penetrance genes (**Supplementary Table 1**, **Figure 2D**).

Regarding the types of variants, 82.3% (56/68) were missense, 13.2% (9/68) were loss-of-function (LoF), 1.5% (1/68) were splicing mutations, and 3% (2/68) were synonymous variants. The genes with the most frequently observed variants were *MSH6* (n=5), and *MLH1, WRN, FANCC, KDR*, and *TCF7L2* (each with 3 variants, respectively) (**Table 3**). Finally, five of the oncodriver variants were novel and had not been previously reported in public databases (*APC*: c.3663_3665delTTC, *MLH1*: c.1039delA, *RAD51C*: c.659T>C, *CTNNB1*: c.991T>C, and *KDR*: c.2555A>T). All these variants were confirmed through Sanger sequencing.

The comparison between allelic frequencies of P/LP variants identified by Filter A and oncodriver variants identified by Filter B revealed statistically significant differences (p<0.05) when compared to those described for global and Latin American populations in the gnomAD database (**Supplementary Table 2**). For P/LP variants classified by Filter A, 46% had higher allelic frequencies compared to the global population, while only 23% differed from the Latin American population. This finding highlights distinct genomic profiles specific to our population, with higher frequencies of P/LP variants associated with CRC.

Similarly, 42.6% of the oncodriver variants identified by Filter B showed significant differences in global allelic frequencies, while 70.6% of the allelic frequencies in our patients aligned with those of the Latin American population (**Supplementary Table 2**).

### *In vitro* assay in splice-site variants

Functional validation was performed for three splicing mutations identified in the genes *ERCC1:* c.702 + 1G>A, *SMAD9:* c.781 + 2T>A, and *IL12RB1:* c.1791 + 2T>G. These variants belong to genes that are not typically included in cancer diagnostic panels, representing novel genetic factors potentially related to CRC.

**Figure 4:**
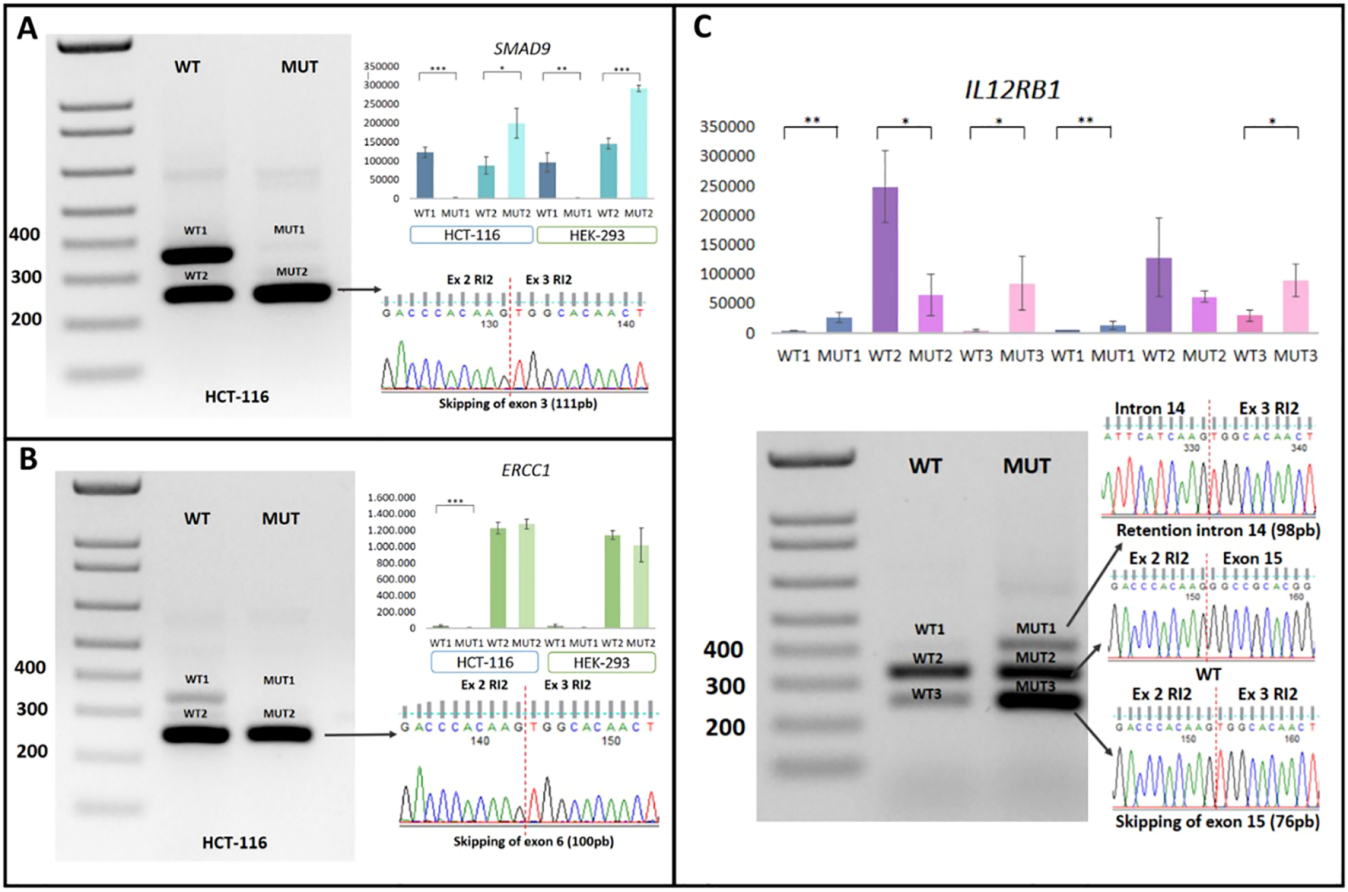
Minigene assay on intronic P/LP variants in canonical splicing sites. **(A)** Functional analysis of *SMAD9*:c.781 + 2T>A in transfected HCT-116 and HEK-293 cells (WT and MUT), with exon skipping confirmed by Sanger sequencing. **(B)** Functional analysis of *ERCC1*:c.702 + 1G>A transfected HCT-116 and HEK-293 cells (WT and MUT), with exon skipping confirmed by Sanger sequencing. **(C)** Functional analysis of *IL12RB1*:c.1791 + 2T>G in transfected HCT-116 and HEK-293 cells (WT and MUT), showing exon skipping and an intron retention confirmed by Sanger sequencing. Ex, exon; MUT, mutant; RI2, Rat Insulin 2; WT, Wild-Type *Statistical significance. *p<0.05; **p<0.01 and ***p<0.001.

Confirmation of the generation of aberrant transcripts, validated through post-transfection RT-PCR in HCT-116 and HEK-293 cell models, revealed that the *SMAD9* c.781 + 2T>A mutation induced exon skipping of exon 3, resulting in the loss of 111 bp. The mutant form failed to generate the normal transcript observed in the WT version, leading to complete exon skipping and the loss of 37 amino acids in the protein (p<0.05). This result was consistent across all three replicates and in both cell lines analyzed. Exon skipping was further confirmed through Sanger sequencing of plasmid DNA obtained from the cloning process (**Figure 4A**).

Functional analysis of the *ERCC1* c.702 + 1G>A variant revealed the exclusive generation of an aberrant transcript with a loss of 100 bp, corresponding to exon 6 skipping, which result in a frameshift mutation (p. Ser201Argfs*1). The wild-type (WT) allele produced two transcripts: one canonical and one resulting from exon skipping. A statistically significant difference in transcript profiles was observed between the WT and mutant (MUT) alleles in the HCT-116 cell line (p < 0.05). This difference was not observed in the HEK-293 Cell line (**Figure 4B**).

Lastly, the *IL12RB1* c.1791 + 2T>G mutation was associated with the generation of two aberrant transcripts. The first involved partial retention of intron 14 of the *IL12RB1* gene, introducing 98 bp, while the second resulted in exon skipping, eliminating exon 15 (76 bp). The predicted consequences on the protein indicate that these transcripts induce frameshift mutations, p.Thr598Glyfs21 and p.A573Lfs21, respectively (**Figure 4C**). Although these aberrant transcripts were also observed in the WT version, their expression was significantly reduced compared to the MUT version (p<0.05). These findings were consistent across both cell models analyzed and in all three replicates. The transcripts were verified through Sanger sequencing of the plasmid DNA obtained from cloning using the TOPO^®^ TA Cloning^®^ Kit for Sequencing (Invitrogen) (**Figure 4C**).

## Discussion

To the best of our knowledge, this study presents, for the first time, a description of the prevalence and spectrum of germline variants in a cohort of Colombian patients with unselected CRC. Our approach utilized next-generation sequencing (NGS) to identify molecular variants within a comprehensive panel of 206 cancer-associated genes (**Table 1**). This panel includes genes commonly used in diagnostic panels for clinical practice, as well as potential candidate genes identified through an exhaustive literature review. The bioinformatic pipeline implemented enabled the classification of molecular variants according to the ACMG/AMP criteria and the BoostDM artificial intelligence model (**Figure 1**).

Although BoostDM was originally developed for somatic variants, we selected this model due to its comprehensive *in silico* saturation mutagenesis approach, which enables a systematic assessment of the oncogenic potential of all possible variants across cancer-related genes (32). Given the lack of germline-specific tools for oncodriver classification, we aimed to explore the applicability of BoostDM to germline variants. To ensure its relevance in this context, we compared its performance with AlphaMissense, which demonstrated strong concordance and suggested potential utility beyond the somatic mutation context (33). Notably, this strategy has previously been explored and validated in several populations worldwide exposed to driver mutations in lung and gastric cancers (35, 36).

Our cohort consisted of unselected patients, with only 12% being diagnosed with CRC before the age of 50. This contrasts with the majority of studies on germline mutations, which typically focus on individuals with early-onset cancer or those suspected of having hereditary predisposition syndromes related to CRC (18, 23, 37–39). In such cases, CRC susceptibility is often primarily attributed to germline variants, particularly in hereditary or syndromic CRC, where inheritance typically follows an autosomal dominant pattern.

In addition to the age of CRC onset, our study revealed a higher proportion of affected women (55%) compared to men, which contrasts with previously reported findings. Most studies show a higher incidence of CRC in men (20, 37, 40, 41). This discrepancy may be related to demographic trends in Colombia, where the proportion of women is slightly higher than men (20.9% vs 19.9%) in the age ranges most represented in our population. It has been established that the only age group in which men outnumber women is the youngest (0 to 14 years) (https://www.statista.com/statistics/789705/population-total-age-gender-colombia/).

Understanding the genetic factors related to CRC is crucial for supporting the implementation of appropriate surveillance strategies, as recommended by international guidelines, to reduce CRC incidence and mortality in carriers of pathogenic/likely pathogenic (P/LP) germline variants (18). In this context, our study proposes the use of NGS for the analysis of multiple genes, enabling the identification of both canonical and non-canonical pathogenic variants associated with the disease. Non-canonical PVs in cancer predisposition genes, seemingly unrelated to CRC, have proven to be a significant source for identifying the molecular causes in unselected cases (21). Moreover, analyzing unselected populations, as we have done, has been recommended to avoid underestimating the true prevalence of hereditary factors in the global CRC population (18).

Regarding the classification of molecular variants, we applied two approaches. First, Filter A, which followed the ACMG/AMP recommendations (30, 42). This approach is used in both research and routine clinical molecular diagnostics, and it enabled the detection of 13 P/LP variants in 12% of our patients (**Table 3**).

Our findings demonstrated a higher detection rate compared to other studies analyzing unselected populations, such as those described by Yurgelun et al. (21), which reported a rate of 10% (21). The smaller number of genes analyzed in that study (n=29) may explain this difference and supports the advantage of using expanded panels, which potentially enhance diagnostic performance (18, 43). Detection rates of PVs in cases with suspected familial CRC or in patients diagnosed before the age of 50 are substantially higher, with rates ranging from 15.5% to 26.8% (18, 20, 40, 41). In our study, 61.5% of the P/LP variants were associated with genes included in diagnostic panels, while the remaining 38.5% originated from candidate genes identified through literature review. This significant proportion supports the hypothesis that the analysis of genes not currently included in routine diagnostic panels on various sequencing platforms can increase the detection of P/LP germline variants associated with CRC development.

In the analyzed population, four high-penetrance mutations were identified in the *MLH1, MSH6, BMPR1A*, and *PTCH1* genes. This finding is significant, as the detection of mutations in some of these genes, particularly *MLH1* and *MSH6*, allows for the implementation of screening, treatment, and follow-up strategies for these high-risk patients, following the recommendations outlined in the NCCN guidelines (National Comprehensive Cancer Network) (https://www.nccn.org/patients/guidelines/content/PDF/colorectal-screening-patient.pdf). Germline variants in the mismatch repair (MMR) genes (*MLH1, MSH2, MSH6*, and *PMS2*) are associated with Lynch Syndrome, one of the most common genetic predisposition syndromes, accounting for 2-4% of all CRC cases. Studies have shown that in Latin American and Caribbean countries, there is a higher prevalence of CRC in patients under 50 years of age. Therefore, screening programs and the identification of high-risk individuals carrying pathogenic germline mutations are expected to more effectively reduce CRC incidence (44).

Previous studies have demonstrated that most P/LP variants found in CRC patients are located in MMR genes, which are associated with DNA repair processes. For instance, Gong et al. (20) reported that out of the 19.1% of P/LP variants identified in their study, 11.3% were from MMR genes. Similarly, Yurgelun et al. (21) demonstrated a high rate of mutations in these genes, and other studies, such as Zhang et al. (39), confirmed these findings, with nearly 50% of genetic variants located in MMR genes (20, 21, 39).

In contrast to these previous findings, our population exhibited only two variants in the MMR genes (*MLH1*: c.1039del and *MSH6*: c.3516_3517delAG), accounting for 15.4% of the total identified. This result indicates a lower frequency of MMR mutations in our study population, which may be attributed to the sample selection criteria. Studies with higher mutation rates in MMR genes typically include patients with a family history of CRC, a diagnosis under the age of 50, and the presence of polyps. In contrast, our study analyzed unselected cases, allowing for the identification of variants in genes not associated with hereditary syndromes.

Additionally, mutations in other genes related to syndromic CRC were identified in our study. One such mutation is the *BMPR1A* mutation (c.176T>A, p.Leu59Ter), which generates a premature termination codon (PTC), leading to the elimination of 473 amino acids. Variants in the *BMPR1A* gene have been associated with juvenile polyposis syndrome. This gene is linked to the AKT signaling pathway and functions as a type 1 receptor in the TGFβ superfamily (45–48). Beyond its role in regulating epithelial functions in the colon*, BMPR1A* influences critical cellular processes such as growth, differentiation, and apoptosis (46, 48–51). Furthermore, this mutation has been previously reported in a patient with adenomatous polyposis (52). The identified mutation affects the majority of the protein, including its functional activin type I and II domains, as well as the Ser/Thr protein kinase domain. As a result, the protein loses its functionality, its ability to interact with other proteins, and its role in the aforementioned signaling pathways.

Finally, one patient presented a heterozygous variant in a gene known for its high penetrance and its correlation with metastatic potential in CRC (53). The identified variant is a missense change in the *PTCH1* gene (c.3241G>A, p.Val1081Met). *PTCH1* plays a crucial role in the Hedgehog signaling pathway, which is involved in tumorigenesis, regulation of proliferation, angiogenesis, and stem cell renewal. These processes have been linked to oncogenic mechanisms related to CRC (54). The discovery of this variant is highly relevant, as previous studies have shown that *PTCH1* could serve as a potential biomarker for distinguishing between CRC cases with high and low metastatic risk, with an inverse correlation to *PTCH1* protein expression levels (53). Additionally, this variant has been reported in patients with hereditary cancer predisposition syndromes, with functional evidence indicating protein loss of function (55), further supporting the pathogenicity criteria for this mutation.

For other P/LP variants identified according to ACMG/AMP recommendations, the exact definition of penetrance is not available. However, due to their pathogenicity and clear involvement in molecular signaling pathways related to the CRC pathophysiology, they are relevant in its molecular etiopathology. 46% of the variants corresponded to loss-of-function (LoF) variants and altered the function of *FLCN, NOTCH3, NTHL1*, and *BARD1* genes.

*BARD1* encodes a protein that interacts with the N-terminal region of the *BRCA1* gene. The *BARD1/BRCA1* complex plays a critical role in DNA repair, recombination, and cell cycle control (56–60). The *BARD1* c.2229dupT variant generates a premature termination codon (PTC), affecting the BCRT 2 domain, which is involved in protein-protein interactions and has been linked to DNA repair, recombination, and cell cycle control—mechanisms critical in the development of malignant neoplasms (61, 62).

On the other hand, the *FLCN* gene is associated with Birt-Hogg-Dubé syndrome and increases the risk of CRC (63). Pathogenic variants in this gene have recently been identified in patients with early-onset CRC (64–68). The *FLCN* c.1285delC variant induces a frameshift (p.His429Thrfs*39), resulting in the loss of the terminal DENN domain, which is important for the AMPK and mTOR signaling pathways (69).

*NOTCH3* is a cell surface receptor that plays a crucial role in signaling pathways that regulate epithelial cell proliferation, polarity/adhesion, and apoptosis (70–72). One patient was a carrier of the *NOTCH3* c.1345C>T, p.Arg449Cys variant, classified as likely pathogenic. Previous research has established a connection between variants in this gene and susceptibility to CRC, particularly in men (73).

The *NTHL1* gene is associated with familial adenomatous polyposis and hereditary cancer predisposition syndrome, with an autosomal recessive inheritance pattern. It is involved in base excision repair (BER), the primary repair pathway for oxidative DNA damage (22, 74–76). The *NTHL1* c.244C>T, p.Gln82Ter variant induces the loss of the HhH domain, which is crucial for DNA binding and, consequently, for the proper functioning of the BER pathway (77).

Our study identified that approximately 40% of the P/LP variants classified through the ACMG/AMP criteria (Filter A described in the methodology) were located in genes not typically analyzed in the context of clinical-molecular diagnosis. Our finding supports the need to implement molecular analyses, using NGS, that allow the simultaneous analysis of hundreds of genes potentially related to CRC. 60% of these variants were located in genomic sites involved in the splicing process and correspond to the *ERCC1* (c.702 + 1G>A), *IL12RB1* (c.1791 + 2T>G), and *SMAD9* (c.781 + 2T>A) mutations. These genes participate in the signaling pathways of cellular damage repair and processes related to carcinogenesis (78–80). Mutations in these genes could, therefore, alter these signaling pathways and increase susceptibility to CRC (81, 82). However, to determine the effect of these intronic variants on carcinogenesis, further studies must be performed at several levels such as transcriptomic, post-transcriptional modifications, and proteomics in the tumoral context. To the best of our knowledge, these studies have not yet been conducted.

Our study also identified heterozygous P/LP mutations in the *EXO1* and *OGG1* genes, both of which are involved in repair and recombination processes (83–88). When these genes are affected by mutations, replication and post-replication processes, including checkpoint activation, are disrupted, potentially leading to genomic instability and cancer development (84, 89, 90).

Collectively, our findings indicate that germline P/LP variants predominantly cluster in genes associated with DNA repair and regulatory pathways, such as *BARD1, BMPR1A, ERCC1, EXO1, MLH1, MSH6, NTHL1*, and *OGG1* (comprising 60% of the identified variants). These pathways are critical for maintaining genomic stability (91). Consequently, germline variants in genes linked to these signaling pathways can have detrimental effects, increasing susceptibility to CRC.

In addition to searching for pathogenic germline variants using strategies commonly employed in routine molecular diagnostics for identifying individuals at high risk of developing CRC, we utilized an artificial intelligence method. BoostDM is a machine learning-based methodology designed for *in silico* mutagenesis of genes associated with cancer development. This innovative approach systematically evaluates all possible changes within a gene or protein to identify cancer-causing factors (32, 92). Variants with detrimental effects are categorized as oncodrivers. We identified a total of 68 oncodriver variants in 65% of the patients, with 27% presenting more than one oncodriver variant. Of these, 72% were found in genes commonly used in clinical diagnostics, while the remaining 28% were in candidate genes.

The application of BoostDM to germline variant analysis addresses a critical gap in current interpretation strategies. Most existing germline-focused tools, including AlphaMissense, are designed to assess pathogenicity but do not distinguish between driver and passenger mutations—a distinction that can provide deeper insight into cancer predisposition mechanisms (33). BoostDM offers a complementary approach by prioritizing variants with high oncogenic potential, regardless of their previous clinical annotation (32). This feature is particularly useful in unselected populations, where novel or non-canonical germline variants are frequently detected. While BoostDM is not intended to replace clinically validated models, its application can improve variant prioritization for downstream functional assays and support comprehensive molecular profiling in hereditary cancer research (**Figure 3**).

A consensus was established to support the efficacy of the BoostDM model in our analysis, we analyzed its predictions with those from AlphaMissense, a tool recognized for its accuracy in classifying pathogenic germline variants (**Figure 3**). This comparison aimed to ascertain BoostDM’s reliability in the germline context, despite its original design for somatic variant analysis. Our data supports the potential applicability of BoostDM beyond its initial somatic mutation focus and suggests its utility for germline variant analysis in cancer genes. These findings, like those with the previous filter, demonstrate enhanced diagnostic performance with the incorporation of massive sequencing methods such as NGS in the analysis of cancer-related mutations. Recently, Garg et al., (93), indicated that the use of NGS increases the identification of mutations by approximately 36% compared to single-gene analysis, highlighting the clear benefit of using massive molecular analysis strategies (93).

Of the total germline variants classified as oncodriver by BoostDM, a higher proportion of changes in genes related to CRC predisposition syndromes was identified compared to filter A. Significantly, 20.6% of molecular variants were found in these genes, and it was possible to identify them in genes related to Lynch Syndrome; *MSH6* (n=5) and *MLH1* (n=3). As mentioned earlier, these findings allow for advising patients and their relatives following international guidelines, potentially improving the early detection of at-risk individuals and the management of carriers of such variants (94). Additionally, this model allowed the identification of other variants in genes related to syndromic CRC, such as *APC, BMPR1A, MSH2, MSH3, NTHL1*, and *PMS2*. These molecular changes, along with those observed in genes related to DNA damage repair signaling pathways, demonstrate the ability of this artificial intelligence algorithm to detect oncodriver variants (76, 85, 86). Variations in these genes have been reported in large cohorts of patients with highly significant associations with the development of CRC, such as *MSH2* (OR: 18.1), *MLH1* (OR: 8.6), and *APC* (OR: 49.4), supporting the importance of germline variant identification studies in an unselected population to contribute to the generation of genetic assessment policies and variant interpretation in CRC (95).

In this context, based on our results, we believe that the oncodriver variants identified particularly those not detected by conventional prediction algorithms hold potential for inclusion in future clinical prediction panels. However, their inclusion requires further validation, and we propose the following next steps: a) replication in larger and independent cohorts of CRC patients, b) functional validation of candidate variants to confirm their biological relevance, and c) integration of these variants into multigene risk models to evaluate their predictive value in clinical practice. Additionally, drawing on international experiences such as the Personalized OncoGenomics (POG) program in Canada (96), it would be necessary to define a framework for identifying, evaluating, and reporting research-based germline findings within the clinical infrastructure of a publicly funded healthcare system. In this context, since the variants identified by BoostDM are potentially oncogenic, prioritization could be given to those associated with moderate to high penetrance cancer susceptibility genes, or variants in cancer predisposition genes known to influence tumor phenotype and evolution.

Recent studies have recognized the involvement of synonymous variants as causal factors in Mendelian and multifactorial diseases, including cancer (97–103). Interestingly, the BoostDM model identified two synonymous variants in the *CDH1* (c.1710T>C) and *NF1* (c.3498C>T) genes as oncodrivers. This finding is particularly intriguing because it involves genes related to neurofibromatosis and hereditary diffuse gastric cancer. The *NF1* gene is a tumor suppressor that contributes to cancer development and has been associated with gastrointestinal tract adenocarcinoma (104). Studies by Seminog and Goldacre (105) determined that patients with pathogenic mutations in the *NF1* gene have a higher risk of colon cancer compared to the general population, supporting our findings in the patient analyzed (105). It is important to note that, although the variant in the *NF1* gene is classified as an oncodriver by Filter B, it does not meet the criteria to be classified as pathogenic or likely pathogenic (P/LP) according to ACMG/AMP guidelines. Therefore, this variant is not reported as associated with neurofibromatosis.

On the other hand, pathogenic germline variants in the *CDH1* gene are responsible for over 20% of hereditary diffuse gastric cancer. However, consistent with our findings, recent studies have established that carrying a variant in this gene generates a higher predisposition to colorectal polyps, suggesting a potential association between *CDH1* variants and CRC risk. These data are relevant for cancer risk assessment and patient counseling with variants in this gene (106). The molecular mechanisms associated with the pathogenicity of synonymous variants have been explored, but it is generally considered that they mostly affect splicing, generating aberrant transcripts (e.g., exon skipping). A recent study analyzing this type of variants in over 3000 cancer samples demonstrated that around 6-8% of all driver variants in oncogenes are synonymous (107). In this context, we can highlight the importance of incorporating additional methods for detecting variants related to cancer development, as ACMG/AMP criteria may exclude these variants. However, the findings must be approached with caution and supported by functional validation studies.

Additionally, the contribution of oncodriver variants in genes usually used in diagnostic panels, combined with extended molecular analysis of candidate genes, has a significant impact, as demonstrated by our results in which variants were identified in *FANCC, KDR*, and *TCF7L2*. FANCC is involved in DNA repair and transcription processes and has been associated with an increased risk of CRC (108–110). On the other hand, *KDR* encodes one of the two receptors for VEGF. This is a type III tyrosine kinase receptor involved in the proliferation, survival, migration, and tubular morphogenesis of endothelial cells and has been associated as a prognostic marker in CRC (111, 112). Finally, *TCF7L2* plays a key role in the Wnt signaling pathway, and variants in this gene have also been found in patients with CRC (113, 114). Taken together, the findings derived from the BoostDM model highlight a high capacity for identifying oncodriver mutations, supporting recent claims about the use of artificial intelligence as a tool that could enhance precision and effectiveness in cancer prediction, diagnosis, and treatment (115).

The population-genetic analysis carried out in our study identified, for the first time in the country and in Latin America, the frequency of pathogenic, likely pathogenic, and oncodriver variants in 206 genes related to CRC. Comparing allele frequencies with data obtained from the gnomAD database allowed us to establish statistically significant differences between the global and Latin American populations. The greatest differences were observed in non-Latin American populations, demonstrating that approximately 46% of the variants identified by us are presented at higher or lower frequencies. In contrast, only 23% to 29% of the variants in this study were different from other Latin American populations. This finding is relevant as it underscores the need for studies like the present one, where genomic characterization is performed in populations typically underrepresented in public databases.

Taken together, the findings of this study suggest the need to conduct expanded analyses in the Colombian population using whole-exome sequencing (WES) or other high-throughput sequencing methods (e.g., whole-genome sequencing) to establish population-specific variant databases. Our results support this recommendation based on the following observations: (a) 13% of the variants identified were classified as pathogenic or likely pathogenic (P/LP), (b) 38.5% of the P/LP variants were found in genes not currently included in standard diagnostic panels, and (c) we propose a novel classification approach capable of identifying oncodriver variants not detected by conventional algorithms.

Finally, we conducted a functional validation analysis of pathogenic variants identified in genes not conventionally involved in molecular diagnostic genetic panels to contribute new knowledge about their potential involvement in CRC etiology. In this context, intronic variants in canonical splicing sites were evaluated using minigene assay. This type of variant is recognized for its potential effect on splicing and the generation of aberrant transcripts through mechanisms such as exon skipping, intron retention, or pseudogene generation (116).

Our findings revealed that the *SMAD9*: c.781 + 2T>A variant leads to exon skipping, resulting in the loss of 37 amino acids. This molecular effect potentially impacts the protein’s function, crucial in the TGFβ signaling pathway. This pathway has been associated with the regulation of pro-oncogenic processes, such as invasion, epithelial-mesenchymal transition, and the promotion of angiogenesis (117). SNPs in this gene have been identified in CRC patients and associated with an increased risk, mainly due to its role in the TGFβ signaling pathway (38, 118–121).

The *ERCC1* c.702 + 1G>A mutation was classified as an oncodriver by BoostDM, and Likely Pathogenic according to the ACMG classification criteria, as detailed in **Table 3** and **Supplementary Table 1**. Based on both classification frameworks, this variant can be considered pathogenic. Furthermore, the ACMG PVS1 criterion supports the hypothesis of a splicing alteration. Our minigene assay results demonstrated exon skipping without the production of a canonical transcript in the HCT116 cell line, consistent with *In Silico* predictions and bioinformatics based classifications. This mutation is predicted to generate a truncated protein (p.Ser201Argfs*1), which lacks the C-terminal HhH2 domain of ERCC1 (residues 220–297) required for dimerization with XPF (122, 123). The ERCC1-XPF complex is a structure-specific endonuclease involved in nucleotide excision repair (NER), interstrand crosslink (ICL) repair, and double-strand break (DSB) repair. Consequently, its impairment significantly compromises DNA repair capacity, increasing the risk of cancer (124).

However, in our functional validation, exon skipping was also observed in the wild-type plasmid, although the canonical transcript remained detectable in HCT116 cell line. This result is difficult to explain, as although *ERCC1* has been reported to produce isoforms through alternative splicing, these transcript variants differ mainly in their untranslated regions or result from intron retention (125). In this context, additional studies may be required to confirm this finding.

The variant in *IL12RB1*: c.1791 + 2T>G had the most significant impact, resulting in the generation of two aberrant transcripts: exon 15 skipping (76 bp), and intron 14 partial retention (98 bp). Both effects caused a reading frame shift and the generation of a premature stop codon, potentially affecting the structural integrity of the protein. *IL12RB1* is an interleukin receptor that plays a role in DNA damage repair pathways (82, 126). Variants in this gene in CRC patients provide convincing evidence of its role in predisposition to this neoplasia (126, 127).

In agreement with recent reports, we confirmed that the pSplice Express vector, used in combination with the Gateway recombination system, is an effective approach for evaluating splicing alterations. Nearly twenty studies in the literature have successfully applied this strategy, reporting relevant findings related to exon skipping, intron retention, cryptic splice sites, among others. Notably, this approach is suitable for assessing mutations in canonical splice sites as well as deep intronic variants, further supporting its technical robustness (121, 126–130).

### Study limitations

The present study has several noteworthy limitations, which could be summarized as technical and biological. The technical limitations are related to the NGS methodology employed, based on short-read sequencing, leading to difficulties in the variant detection in exons with high GC content, highly homologous, repetitive, or low complexity regions, and pseudogenes resulting in false-positive or false-negative variant calls due to inaccurate sequence alignment and variant calling in these challenging regions (128–131). Another technical limitation correlates with the design of the exome probes and the use of the exome capture kits. It has been reported that they can lead to biases in variant detection due to uneven coverage of some coding regions, resulting in systematic insufficiencies in sequencing depth for certain genes, leading to false negative pathogenic variant calls (132, 133).

The NGS methodology employed falls short in identifying pathogenic variants within deep promoter or intronic regions. Furthermore, this study did not investigate Copy Number Variations (CNVs), which have been documented as potential causative factors in Colorectal Cancer (CRC). Concerning the biological limitations, we assessed germline variants from peripheral blood from a cohort of Colombian patients with unselected CRC. This methodology only allowed us to evaluate the genomics of a bigger landscape that requires a multi-omic approach involving transcriptomics, proteomics, and metabolomics to better understand CRC and the correlation between germline and somatic variants in this intricate network (134–136). Functional validation studies focused on splicing variants’ impact on mRNA, yet protein validations were not conducted. It is also critical to recognize that, while the concordance analysis between BoostDM and AlphaMissense provided insights into BoostDM’s applicability to germline variants, both models have inherent limitations in predicting variant pathogenicity. Further study is required to determine functional consequences of the identified variants. Specifically, the reliance on computational predictions without functional validation may not capture the complete biological impact of certain variants. The BoostDM artificial intelligence analysis also identified variants in genes with undetermined penetrance, necessitating cautious interpretation. Additionally, no functional validation was conducted for synonymous variants proposed as oncodrivers.

We performed a minigene assay, a widely used methodology to evaluate the effect of intronic variants on splicing. This assay allows assessment of the variant’s impact on splicing; however, its potential oncogenic effect can be further investigated using complementary approaches such as proliferation, invasion, and apoptosis assays, among others.

## Conclusion

In conclusion, our findings played a crucial role in delineating the germline mutational landscape among unselected CRC patients within the Colombian population, using a comprehensive multigene panel that includes genes from both established diagnostic panels and candidate genes. The importance of evaluating genes typically omitted from routine diagnostic procedures was evident, shedding light on the potential oversight of a substantial proportion of P/LP variants. This omission is primarily due to the limited availability of information regarding their association with CRC. Therefore, expanding the number of genes potentially related to the etiology of the disease could help us to understand how germline variants contribute to increased susceptibility to CRC. Understanding the genetic predisposition for CRC is essential for early diagnosis, prevention, and patient treatment. While improvements in sequencing technologies and the emergence of advanced artificial intelligence bioinformatics platforms, such as the BoostDM model, have expanded our tools for understanding the mechanisms by which variants affect genes or proteins, including synonymous variants that are often not considered pathogenic, it is necessary to further support the molecular involvement with functional validation analyses.

## Data Availability

The raw data supporting the conclusions of this article will be made available by the authors, without undue reservation.
