# Supplementary material for: Integrating next-generation sequencing and artificial intelligence for the identification and validation of pathogenic variants in colorectal cancer
Source: Front Oncol. 2025 May 19;15:1568205. doi: 10.3389/fonc.2025.1568205 (PMC12127813; doi:10.3389/fonc.2025.1568205)
Supplement: Supplementary file 1 [file DataSheet1.pdf]

""

Script Name: boostdm_alphamissense_comparison.py

Description:

This script automates the process of comparing BoostDM Insilico saturation predictions against AlphaMissense annotations to evaluate their agreement. It involves appending AlphaMissense annotations to BoostDM predictions, generating Precision-Recall curves, and optionally producing graphical representations of these curves.

Prerequisites:

1. Download 'AlphaMissense_hg38.tsv.gz' from https://storage.googleapis.com/dm_alphamissense/AlphaMissense_hg38.tsv.gz. Unzip and rename this file to 'alphamissense_predictions.tsv' and place it in a known directory.

2. Download 'Insilico-saturation.zip' from https://www.intogen.org/boostdm/downloads, which contains BoostDM Insilico saturation prediction files. Extract this zip file into a directory. The script expects .tsv or .tsv.gz files structured in potentially nested directories.

Usage:

python boostdm_alphamissense_comparison.py <directory_path_to_BoostDM_files> <path_to_alphamissense_predictions.tsv> [--make-graphs]

- <directory_path_to_BoostDM_files> is the directory containing extracted BoostDM Insilico saturation files (.tsv or .tsv.gz).

- <path_to_alphamissense_predictions.tsv> is the full path to the 'alphamissense_predictions.tsv' file.

- The optional '--make-graphs' flag triggers the generation of PR curve graphs for each comparison.

Output:

The script outputs annotated BoostDM files with AlphaMissense annotations appended, a summary CSV file containing AUC scores for the PR curves of all processed files, and optionally, PR curve graphs. These outputs are saved in an 'output' subdirectory within the specified BoostDM file directory.

Note: The script recursively searches for .tsv and .tsv.gz files in the given directory, unzips any .tsv.gz files found, and performs the analysis on all .tsv files located.

```
"""


import pandas as pd

from sklearn.metrics import precision_recall_curve, auc

import sys

import os

import numpy as np

import gzip

import shutil

import matplotlib.pyplot as plt

import seaborn as sns


def ensure_dir(file_path):

    directory = os.path.dirname(file_path)

    if not os.path.exists(directory):

        os.makedirs(directory)


def unzip_files(directory_path):
```

```
    """Recursively search for .tsv and .tsv.gz files, unzip the latter, and return a list of all
.tsv file paths."""
    tsv_files = []
    for root, dirs, files in os.walk(directory_path):
        for file in files:
            if file.endswith(".tsv"):
                tsv_path = os.path.join(root, file)
                tsv_files.append(tsv_path)
            elif file.endswith(".tsv.gz"):
                gz_path = os.path.join(root, file)
                tsv_path = gz_path.rsplit('.', 2)[0] + '.tsv'  # Ensure we get the correct .tsv
filename
                with gzip.open(gz_path, 'rb') as f_in, open(tsv_path, 'wb') as f_out:
                    shutil.copyfileobj(f_in, f_out)
                os.remove(gz_path)  # Optionally remove the .gz file after extraction
                tsv_files.append(tsv_path)
    return tsv_files


def create_key_file(input_file, key_file_path):
    ensure_dir(key_file_path)
    with open(input_file, 'r') as infile, open(key_file_path, 'w') as keyfile:
        for line in infile:
            parts = line.strip().split('\t')
            if len(parts) < 5:
                continue
            key = '_'.join([parts[3], parts[4], parts[6]])
            keyfile.write(key + '\n')
```

```
def append_am_info(smaller_file, alphamissense_predictions, key_file_path,
output_file):
    keys = set(open(key_file_path, 'r').read().splitlines())
    am_info_dict = {}
    matched = False
    with open(alphamissense_predictions, 'r') as lfile:
        for line in lfile:
            parts = line.strip().split('\t')
            if len(parts) < 10:
                continue
            key = '_'.join([parts[0].lstrip('chr'), parts[1], parts[7]])
            if key in keys:
                am_info_dict[key] = parts[8:10]
                matched = True

    if not matched:
        print(f"No AlphaMissense predictions matched for
{os.path.basename(smaller_file)}. Skipping.")
        return False

    with open(smaller_file, 'r') as sfile, open(output_file, 'w') as ofile:
        for line in sfile:
            parts = line.strip().split('\t')
            key = '_'.join([parts[3], parts[4], parts[6]])
            am_info = am_info_dict.get(key, ['NA', 'NA'])
            ofile.write(line.strip() + '\t' + '\t'.join(am_info) + '\n')
```

```
    return True


def process_file_for_PR(file_path, output_directory):
    data = pd.read_csv(file_path, sep='\t', low_memory=False)
    data = data[data['NA.1'] != 'NA']
    data['alpha_missense_binary'] = (data['NA.1'] != 'likely_benign').astype(int)
    scores = data['boostDM_score'].values
    true_labels = data['alpha_missense_binary'].values
    precision, recall, _ = precision_recall_curve(true_labels, scores)
    auc_score = auc(recall, precision)
    pr_filename = os.path.basename(file_path).replace('.tsv', '_precision_recall.csv')
    pr_full_path = os.path.join(output_directory, pr_filename)
    ensure_dir(pr_full_path)
    pd.DataFrame({'Precision': precision, 'Recall': recall}).to_csv(pr_full_path,
index=False)
    return auc_score, pr_filename


def make_graph(filename):
    data = pd.read_csv(filename)
    sns.set(style="whitegrid")
    plt.figure(figsize=(8, 6))
    sns.lineplot(data=data, x='Recall', y='Precision', marker='o', linestyle='-')
    plt.title('Precision-Recall Curve')
    plt.xlabel('Recall')
    plt.ylabel('Precision')
    plt.xlim([0, 1])
```

```
    plt.ylim([0.5, 1])

    plt.tight_layout()

    output_filename = filename.replace('.csv', '_pr_curve.png')

    plt.savefig(output_filename)

    plt.close()

    print(f"Graph saved as {output_filename}")


def main(directory_path, alphamissense_predictions, make_graphs=False):

    tsv_files = unzip_files(directory_path)

    output_directory = os.path.join(directory_path, "output")

    ensure_dir(output_directory)

    auc_results = []  # List to store AUC results for each file

    for smaller_file in tsv_files:

        key_file_path = os.path.join(output_directory, os.path.basename(smaller_file) +
'_keys.txt')

        annotated_file = os.path.join(output_directory,
os.path.basename(smaller_file).replace('.tsv', '_annotated.tsv'))

        create_key_file(smaller_file, key_file_path)


        if append_am_info(smaller_file, alphamissense_predictions, key_file_path,
annotated_file):

            auc_score, pr_filename = process_file_for_PR(annotated_file, output_directory)

            print(f"Processed {os.path.basename(smaller_file)}: AUC = {auc_score}")

            if make_graphs:

                make_graph(os.path.join(output_directory, pr_filename))


            auc_results.append({'Filename': pr_filename, 'AUC Score': auc_score})
```

```
    # Save summary of AUC results to a CSV file
    results_df = pd.DataFrame(auc_results)
    summary_path = os.path.join(output_directory, 'results_summary.csv')
    results_df.to_csv(summary_path, index=False)
    print(f"Summary of AUC results saved to {summary_path}")


if __name__ == "__main__":
    if len(sys.argv) < 3 or len(sys.argv) > 4:
        print("Usage: python boostdm_alphamissense_comparison.py <directory_path>
<alphamissense_predictions> [--make-graphs]")
        sys.exit()


    directory_path = sys.argv[1]
    alphamissense_predictions = sys.argv[2]
    make_graphs_flag = '--make-graphs' in sys.argv


    main(directory_path, alphamissense_predictions, make_graphs_flag)
```
