# Supplementary material for: Integrating next-generation sequencing and artificial intelligence for the identification and validation of pathogenic variants in colorectal cancer
Source: Front Oncol. 2025 May 19;15:1568205. doi: 10.3389/fonc.2025.1568205 (PMC12127813; doi:10.3389/fonc.2025.1568205)
Supplement: Supplementary file 2 [file Table1.docx]

**Supplementary Table 1.** Described oncodriver germline variants in CRC patients – Filter B (BoostDM)

| Penetrance | Panel | Gene | Transcript | Variant | Protein | rs ID | Type | Count |
| --- | --- | --- | --- | --- | --- | --- | --- | --- |
| High | Diagnosis | *APC* | NM_000038.6 | c.3663_3665delTTC | p.Ser1223del | Not reported | LOF | 1 |
| High | Diagnosis | *BMPR1A* | NM_004329.3 | c.176T>A | p.Leu59Ter | rs1564714834 | LOF | 1 |
| High | Diagnosis | *BRCA2* | NM_000059.4 | c.8851G>A | p.Ala2951Thr | rs11571769 | Missense | 11 |
| High | Diagnosis | *CDH1* | NM_004360.5 | c.1710T>C | p.Asn570= | rs202115589 | Sinónima | 1 |
| High | Diagnosis | *MLH1* | NM_000249.4 | c.1039delA | p.Thr347Leufs*20 | Not reported | LOF | 1 |
| High | Diagnosis | *MLH1* | NM_000249.4 | c.1514G>A | p.Ser505Asn | rs771044689 | Missense | 1 |
| High | Diagnosis | *MLH1* | NM_000249.4 | c.1852A>G | p.Lys618Glu | rs35001569 | Missense | 1 |
| High | Diagnosis | *MSH2* | NM_000251.3 | c.128A>G | p.Tyr43Cys | rs17217723 | Missense | 1 |
| High | Diagnosis | *MSH3* | NM_002439.5 | c.1622C>T | p.Thr541Ile | rs1178614269 | Missense | 1 |
| High | Diagnosis | *MSH6* | NM_000179.3 | c.3516_3517delAG | p.Arg1172Serfs*4 | rs398123232 | LOF | 1 |
| High | Diagnosis | *MSH6* | NM_000179.3 | c.1403G>A | p.Arg468His | rs41295268 | Missense | 1 |
| High | Diagnosis | *MSH6* | NM_000179.3 | c.2041C>T | p.Leu681Phe | rs1553413412 | Missense | 1 |
| High | Diagnosis | *MSH6* | NM_000179.3 | c.3961A>G | p.Arg1321Gly | rs41295278 | Missense | 1 |
| High | Diagnosis | *MSH6* | NM_000179.3 | c.3245C>T | p.Pro1082Leu | rs191109849 | Missense | 1 |
| High | Diagnosis | *NTHL1* | NM_002528.7 | c.244C>T | p.Gln82Ter | rs150766139 | LOF | 1 |
| High | Diagnosis | *PALB2* | NM_024675.4 | c.2794G>A | p.Val932Met | rs45624036 | Missense | 1 |
| High | Diagnosis | *PMS2* | NM_000535.7 | c.2395C>T | p.Arg799Trp | rs149202766 | Missense | 2 |
| High | Diagnosis | *POLD1* | NM_002691.4 | c.2052G>C | p.Gln684His | rs144143245 | Missense | 1 |
| High | Diagnosis | *POLD1* | NM_002691.4 | c.2017G>A | p.Glu673Lys | rs61751955 | Missense | 1 |
| High | Diagnosis | *POLE* | NM_006231.4 | c.686A>C | p.His229Pro | rs1482513360 | Missense | 1 |
| High | Diagnosis | *POLE* | NM_006231.4 | c.6494G>A | p.Arg2165His | rs5745068 | Missense | 1 |
| High | Diagnosis | *PTCH1* | NM_000264.5 | c.3241G>A | p.Val1081Met | rs587778629 | Missense | 1 |
| High | Diagnosis | *PTCH1* | NM_000264.5 | c.1808G>A | p.Arg603His | rs199523893 | Missense | 1 |
| High | Diagnosis | *PTCH1* | NM_000264.5 | c.3487G>A | p.Gly1163Ser | rs113663584 | Missense | 1 |
| Moderate | Diagnosis | *ATM* | NM_000051.4 | c.6313A>G | p.Arg2105Gly | rs879253983 | Missense | 2 |
| Moderate | Diagnosis | *ATM* | NM_000051.4 | c.7502A>G | p.Asn2501Ser | rs531617441 | Missense | 1 |
| Moderate | Diagnosis | *BARD1* | NM_000465.2 | c.1075_1095del | p.Leu359_Pro365del | rs28997575 | LOF | 3 |
| Moderate | Diagnosis | *CHEK2* | NM_001005735.2 | c.1556C>T | p.Thr519Met | rs142763740 | Missense | 1 |
| Moderate | Diagnosis | *RAD51C* | NM_058216.3 | c.659T>C | p.Leu220Pro | Not reported | Missense | 1 |
| Moderate | Diagnosis | *RAD51C* | NM_058216.3 | c.859A>G | p.Thr287Ala | rs28363317 | Missense | 3 |
| Less well-defined | Diagnosis | *BLM* | NM_000057.4 | c.2452C>T | p.Arg818Cys | rs1279814185 | Missense | 1 |
| Less well-defined | Diagnosis | *BLM* | NM_000057.4 | c.2594A>G | p.Tyr865Cys | rs777842626 | Missense | 1 |
| Less well-defined | Diagnosis | *BRIP1* | NM_032043.3 | c.517C>T | p.Arg173Cys | rs4988345 | Missense | 2 |
| Less well-defined | Diagnosis | *EGFR* | NM_005228.5 | c.2380C>A | p.Pro794Thr | rs370289230 | Missense | 1 |
| Less well-defined | Diagnosis | *FH* | NM_000143.4 | c.535G>A | p.Val179Ile | rs1553341588 | Missense | 1 |
| Less well-defined | Diagnosis | *FLCN* | NM_144997.7 | c.1285delC | p.His429Thrfs*39 | rs80338682 | LOF | 1 |
| Less well-defined | Diagnosis | *FLCN* | NM_144997.7 | c.535C>T | p.Arg179Trp | rs774358971 | Missense | 1 |
| Less well-defined | Diagnosis | *GPC3* | NM_004484.4 | c.1285G>A | p.Val429Met | rs11539789 | Missense | 3 |
| Less well-defined | Diagnosis | *NBN* | NM_002485.5 | c.643C>T | p.Arg215Trp | rs34767364 | Missense | 1 |
| Less well-defined | Diagnosis | *NF1* | NM_000267.3 | c.3498C>T | p.Gly1166= | rs2066733 | Synonymous | 1 |
| Less well-defined | Diagnosis | *PDGFRA* | NM_006206.6 | c.2282T>G | p.Leu761Arg | rs148654387 | Missense | 4 |
| Less well-defined | Diagnosis | *PHOX2B* | NM_003924.4 | c.738_776del | p.Ala248_Ala260del | rs757020181 | Missense | 1 |
| Less well-defined | Diagnosis | *PMS1* | NM_000534.5 | c.329C>G | p.Thr110Arg | rs372752293 | Missense | 1 |
| Less well-defined | Diagnosis | *RAD50* | NM_005732.4 | c.113A>G | p.Asn38Ser | rs750480943 | Missense | 1 |
| Less well-defined | Diagnosis | *RAD50* | NM_005732.4 | c.572C>T | p.Thr191Ile | rs2230017 | Missense | 1 |
| Less well-defined | Diagnosis | *TSC2* | NM_000548.5 | c.2161G>A | p.Val721Met | rs768261185 | Missense | 1 |
| Less well-defined | Diagnosis | *WRN* | NM_000553.6 | c.1717A>G | p.Thr573Ala | rs150148567 | Missense | 1 |
| Less well-defined | Diagnosis | *WRN* | NM_000553.6 | c.1192A>G | p.Thr398Ala | rs532554615 | Missense | 1 |
| Less well-defined | Diagnosis | *WRN* | NM_000553.6 | c.2500C>T | p.Arg834Cys | rs3087425 | Missense | 3 |
| Less well-defined | Candidate | *CRTC3* | NM_022769.5 | c.1789A>C | p.Met597Leu | rs777546566 | Missense | 1 |
| Less well-defined | Candidate | *CTNNB1* | NM_001904.4 | c.991T>C | p.Tyr331His | Not reported | Missense | 1 |
| Less well-defined | Candidate | *ERCC1* | NM_202001.3 | c.702+1G>A | - | rs747911302 | Splice | 1 |
| Less well-defined | Candidate | *ERCC1* | NM_202001.3 | c.875G>A | p.Trp292Ter | rs116640350 | LOF | 1 |
| Less well-defined | Candidate | *FANCC* | NM_000136.3 | c.851C>T | p.Ala284Val | rs201281511 | Missense | 2 |
| Less well-defined | Candidate | *FANCC* | NM_000136.3 | c.77C>T | p.Ser26Phe | rs1800361 | Missense | 3 |
| Less well-defined | Candidate | *FANCC* | NM_000136.3 | c.584A>T | p.Asp195Val | rs1800365 | Missense | 1 |
| Less well-defined | Candidate | *FUT2* | NM_000511.6 | c.412C>T | p.Arg138Cys | rs1800022 | Missense | 2 |
| Less well-defined | Candidate | *FUT2* | NM_000511.6 | c.812del | p.Pro271Leufs*16 | rs1799761 | LOF | 1 |
| Less well-defined | Candidate | *KDR* | NM_002253.4 | c.2555A>T | p.Asp852Val | Not reported | Missense | 1 |
| Less well-defined | Candidate | *KDR* | NM_002253.4 | c.2837G>A | p.Arg946His | rs140041720 | Missense | 1 |
| Less well-defined | Candidate | *KDR* | NM_002253.4 | c.2312C>T | p.Thr771Met | rs149745504 | Missense | 1 |
| Less well-defined | Candidate | *MRE11* | NM_005591.4 | c.2048G>A | p.Gly683Glu | rs764705257 | Missense | 1 |
| Less well-defined | Candidate | *SH2B3* | NM_005475.3 | c.232G>A | p.Glu78Lys | rs754838420\| | Missense | 1 |
| Less well-defined | Candidate | *TBX3* | NM_005996.4 | c.613C>T | p.His205Tyr | rs749487839 | Missense | 1 |
| Less well-defined | Candidate | *TCF7L2* | NM_030756.5 | c.1466C>G | p.Pro489Arg | rs77673441 | Missense | 2 |
| Less well-defined | Candidate | *TCF7L2* | NM_030756.5 | c.1466C>A | p.Pro489His | rs77673441 | Missense | 5 |
| Less well-defined | Candidate | *TCF7L2* | NM_030756.5 | c.1472C>T | p.Pro491Leu | rs573425555 | Missense | 1 |
| Less well-defined | Candidate | *TP53BP1* | NM_005657.4 | c.5482C>T | p.His1828Tyr | rs780546857 | Missense | 1 |

Abbreviations: LOF, Loss of function.
