# Supplementary material for: Integrating next-generation sequencing and artificial intelligence for the identification and validation of pathogenic variants in colorectal cancer
Source: Front Oncol. 2025 May 19;15:1568205. doi: 10.3389/fonc.2025.1568205 (PMC12127813; doi:10.3389/fonc.2025.1568205)
Supplement: Supplementary file 3 [file Table2.docx]

**Supplementary Table 2.** Pathogenic/likely pathogenic and oncodriver variants allelic frequencies comparison

| **Filter A - ACMG/AMP Classification** | | | | | | | | | |
| --- | --- | --- | --- | --- | --- | --- | --- | --- | --- |
| **Gene** | **Variant** | **rs ID** | **Allele frequencies** | **gnomAD general population allele frequencies** | **p-value** | **p-value yates’ correction** | **gnomAD latin allele frequencies** | **p-value** | **p-value Yates correction** |
| *BARD1* | c.2229dupT | rs1259296823 | 0,005 | 3,97902E-06 | < 0.00001 | < 0.00001* | 2,89218E-05 | < 0.00001 | < 0.00001* |
| *BMPR1A* | c.176T>A | rs1564714834 | 0,005 | NR | NA | NA | NR | NA | NA |
| *ERCC1* | c.702+1G>A | rs747911302 | 0,005 | 3,97763E-06 | < 0.00001 | < 0.00001* | NR | NA | NA |
| *EXO1* | c.1465delA | Not reported | 0,005 | NR | NA | NA | NR | NA | NA |
| *FLCN* | c.1285delC | rs80338682 | 0,005 | 1,22287E-05 | < 0.00001 | < 0.00001* | NR | NA | NA |
| *IL12RB1* | c.1791+2T>G | rs554063682 | 0,005 | 0,000102081 | < 0.00001 | < 0.00001* | 0,000120034 | < 0.00001 | < 0.00001* |
| *MLH1* | c.1039delA | Not reported | 0,005 | NR | NA | NA | NR | NA | NA |
| *MSH6* | c.3516_3517delAG | rs398123232 | 0,005 | NR | NA | NA | NR | NA | NA |
| *NOTCH3* | c.1345C>T | rs762734007 | 0,005 | NR | NA | NA | NR | NA | NA |
| *NTHL1* | c.244C>T | rs150766139 | 0,005 | 0,001417324 | 0,178 | 0,684 | 0,000705577 | 0,0248 | 0,3523 |
| *OGG1* | c.137G>A | rs104893751 | 0,005 | 0,002165888 | 0,388476 | 0,919451 | 0,001864091 | 0,31 | 0,84 |
| *PTCH1* | c.3241G>A | rs587778629 | 0,005 | 9,15182E-05 | < 0.00001 | < 0.00001* | 0,000260191 | 0,00008 | 0,06 |
| *SMAD9* | c.781+2T>A | rs770716081 | 0,005 | 7,98433E-05 | < 0.00001 | < 0.00001* | 3,91942E-05 | < 0.00001 | < 0.00001* |
| **Filter B - BoostDM Classification** | | | | | | | | | |
| **Gen** | **Variant** | **rs ID** | **Allele frequencies^b^** | **gnomAD general allele frequencies** | **p-value** | **p-value yates correction** | **gnomAD latin allele frequencies** | **p-value** | **p-value Yates correction** |
| *APC* | c.3663_3665delTTC | NR | 0,005 | - | NA | NA | - | NA | NA |
| *ATM* | c.6313A>G | rs879253983 | 0,010 | 0,0000 | < 0.00001 | < 0.00001* | - | NA | NA |
| *ATM* | c.7502A>G | rs531617441 | 0,005 | 0,0002 | < 0.00001 | < 0.00001* | 0,0001 | < 0.00001 | < 0.00001* |
| *BARD1* | c.1075_1095del | rs28997575 | 0,015 | 0,0283 | 0,2665 | 0,3722 | 0,033728814 | 0,143 | 0,206 |
| *BLM* | c.2452C>T | rs1279814185 | 0,005 | 0,0000 | < 0.00001 | < 0.00001* | 0,0000 | < 0.00001 | < 0.00001* |
| *BLM* | c.2594A>G | rs777842626 | 0,010 | 0,0000 | < 0.00001 | < 0.00001* | - | NA | NA |
| *BMPR1A* | c.176T>A | rs1564714834 | 0,005 | - | NA | NA | - | NA | NA |
| *BRCA2* | c.8851G>A | rs11571769 | 0,060 | 0,0089 | < 0.00001 | < 0.00001* | 0,0378 | 0,1689 | 0,2363 |
| *BRIP1* | c.517C>T | rs4988345 | 0,010 | 0,0026 | 0,5140 | 0,9698 | 0,0008 | 0,0468 | 0,4327 |
| *CDH1* | c.1710T>C | [rs202115589](http://www.ncbi.nlm.nih.gov/projects/SNP/snp_ref.cgi?rs=rs202115589) | 0,005 | 0,0000 | < 0.00001 | < 0.00001* | 0,0001 | < 0.00001 | < 0.00001* |
| *CHEK2* | c.1556C>T | rs142763740 | 0,005 | 0,0003 | 0,0002 | 0,0824 | 0,0002 | < 0.00001 | < 0.00001* |
| *CRTC3* | c.1789A>C | rs777546566 | 0,005 | 0,0000 | < 0.00001 | < 0.00001* | 0,0000 | < 0.00001 | < 0.00001* |
| *CTNNB1* | c.991T>C | NR | 0,005 | - | NA | NA | - | NA | NA |
| *EGFR* | c.2380C>A | rs370289230 | 0,005 | - | NA | NA | - | NA | NA |
| *ERCC1* | c.702+1G>A | rs747911302 | 0,005 | 3,97763E-06 | < 0.00001 | < 0.00001* | NR | NA | NA |
| *ERCC1* | c.875G>A | rs116640350 | 0,005 | 0,0026 | 0,4989 | 9,8245 | 0,0021 | 0,0000 | 0,0507 |
| *FANCC* | c.851C>T | rs201281511 | 0,010 | 0,0000 | < 0.00001 | < 0.00001* | 0,0000 | < 0.00001 | < 0.00001* |
| *FANCC* | c.77C>T | rs1800361 | 0,005 | 0,0049 | 0,9774 | 0,6302 | 0,0041 | 0,3563 | 0,8890 |
| *FANCC* | c.584A>T | rs1800365 | 0,005 | 0,0030 | 0,6012 | 0,8994 | 0,0027 | 0,5206 | 0,9633 |
| *FH* | c.535G>A | rs1553341588 | 0,005 | - | NA | NA | - | NA | NA |
| *FLCN* | c.535C>T | rs774358971 | 0,005 | 0,0001 | < 0.00001 | < 0.00001* | 0,0001 | < 0.00001 | < 0.00001* |
| *FLCN* | c.1285delC | rs80338682 | 0,005 | 1,22287E-05 | < 0.00001 | < 0.00001* | - | NA | NA |
| *FUT2* | c.412C>T | rs1800022 | 0,010 | 0,0061 | 0,8472 | 0,7912 | 0,0049 | 0,9855 | 0,6239 |
| *FUT2* | c.812delC | rs1799761 | 0,005 | 0,0015 | 0,1953 | 0,7077 | 0,0007 | 0,0248 | 0,3522 |
| *GPC3* | c.1285G>A | rs11539789 | 0,020 | 0,0059 | 0,0887 | 0,2160 | 0,0204 | 0,6015 | 0,7896 |
| *KDR* | c.2555A>T | NR | 0,005 | - | NA | NA | - | NA | NA |
| *KDR* | c.2837G>A | rs140041720 | 0,005 | 0,0002 | 0,0000 | 0,0410* | 0,0002 | < 0.00001 | < 0.00001* |
| *KDR* | c.2312C>T | rs149745504 | 0,005 | 0,0012 | 0,1282 | 0,6080 | 0,0021 | 0,3780 | 0,9108 |
| *MLH1* | c.1039delA | NR | 0,005 | - | NA | NA | - | NA | NA |
| *MLH1* | c.1514G>A | rs771044689 | 0,005 | 0,0000 | < 0.00001 | < 0.00001* | 0,0001 | < 0.00001 | < 0.00001* |
| *MLH1* | c.1852A>G | rs35001569 | 0,005 | 0,0034 | 0,7020 | 0.822 | 0,0045 | 0.8984 | 0.68 |
| *MRE11* | c.2048G>A | rs764705257 | 0,005 | 0,0000 | < 0.00001 | < 0.00001* | 0,0002 | < 0.00001 | < 0.00001* |
| *MSH2* | c.128A>G | rs17217723 | 0,005 | 0,0001 | < 0.00001 | < 0.00001* | - | NA | NA |
| *MSH3* | c.1622C>T | rs1178614269 | 0,005 | - | NA | NA | - | NA | NA |
| *MSH6* | c.3516_3517delAG | rs398123232 | 0,005 | NR | NA | NA | NR | NA | NA |
| *MSH6* | c.1403G>A | rs41295268 | 0,005 | 0,0000 | < 0.00001 | < 0.00001* | 0,0001 | < 0.00001 | < 0.00001* |
| *MSH6* | c.2041C>T | rs1553413412 | 0,005 | - | NA | NA | - | NA | NA |
| *MSH6* | c.3961A>G | rs41295278 | 0,005 | 0,0001 | < 0.00001 | < 0.00001* | 0,0001 | < 0.00001 | < 0.00001* |
| *MSH6* | c.3245C>T | rs191109849 | 0,005 | 0,0003 | 0,0002 | 0,0811 | 0,0014 | 0,1881 | 0,6980 |
| *NBN* | c.643C>T | rs34767364 | 0,005 | 0,0025 | 0,4630 | 0,9860 | 0,0016 | 0,2362 | 0,7600 |
| *NF1* | c.3498C>T | [rs2066733](http://www.ncbi.nlm.nih.gov/projects/SNP/snp_ref.cgi?rs=rs2066733) | 0,005 | 0,0012 | 0,1220 | 0,5988 | 0,0006 | 0,0123 | 0,2800 |
| *NTHL1* | c.244C>T | rs150766139 | 0,005 | 0,001417324 | 0,178 | 0,684 | 0,000705577 | 0,0248 | 0,3523 |
| *PALB2* | c.2794G>A | rs45624036 | 0,005 | 0,0051 | 0,9800 | 0,6360 | 0,0020 | 0,3466 | 0,8799 |
| *PDGFRA* | c.2282T>G | rs148654387 | 0,020 | 0,0021 | < 0.00001 | < 0.00001* | 0,0159 | 0,6200 | 0,8350 |
| *PHOX2B* | c.738_776del | rs757020181 | 0,010 | 0,0020 | 0,2580 | 0,7860 | 0,0366 | 0,0410 | 0,0690 |
| *PMS1* | c.329C>G | rs372752293 | 0,010 | 0,0001 | < 0.00001 | < 0.00001* | 0,0002 | 0,0000 | 0,04811* |
| *PMS2* | c.2395C>T | rs149202766 | 0,010 | 0,0005 | 0,0043 | 0,2040 | 0,0031 | 0,6230 | 0,8807 |
| *POLD1* | c.2052G>C | rs144143245 | 0,005 | 0,0004 | 0,0021 | 0,1648 | 0,0003 | 0,0006 | 0,1129 |
| *POLD1* | c.2017G>A | rs61751955 | 0,005 | 0,0001 | < 0.00001 | < 0.00001* | 0,0002 | 0,0000 | 0,04488* |
| *POLE* | c.686A>C | rs1482513360 | 0,005 | 0,0000 | < 0.00001 | < 0.00001* | 0,0000 | < 0.00001 | < 0.00001* |
| *POLE* | c.6494G>A | rs5745068 | 0,005 | 0,0000 | < 0.00001 | < 0.00001* | 0,0000 | < 0.00001 | < 0.00001* |
| *PTCH1* | c.1808G>A | rs199523893 | 0,005 | 0,0001 | < 0.00001 | < 0.00001* | 0,0004 | 0,0010 | 0,1310 |
| *PTCH1* | c.3241G>A | rs587778629 | 0,005 | 9,15182E-05 | < 0.00001 | < 0.00001* | 0,000260191 | 0,00008 | 0,06 |
| *PTCH1* | c.3487G>A | rs113663584 | 0,005 | 0,0005 | 0,0052 | 0,2160 | 0,0006 | 0,0099 | 0,2640 |
| *RAD50* | c.113A>G | rs750480943 | 0,005 | 0,0000 | < 0.00001 | < 0.00001* | - | NA | NA |
| *RAD50* | c.572C>T | rs2230017 | 0,005 | 0,0018 | 0,2700 | 0,7980 | 0,0008 | 0,0330 | 0,3860 |
| *RAD51C* | c.659T>C | NR | 0,005 | - | NA | NA | - | NA | NA |
| *RAD51C* | c.859A>G | rs28363317 | 0,015 | 0,0056 | 0,0750 | 0,1919 | 0,0059 | 0,0900 | 0,2190 |
| *SH2B3* | c.232G>A | rs754838420 | 0,005 | 0,0011 | 0,0910 | 0,5400 | 0,0001 | < 0.00001 | < 0.00001* |
| *TBX3* | c.613C>T | rs749487839 | 0,005 | - | NA | NA | - | NA | NA |
| *TCF7L2* | c.1472C>T | rs573425555 | 0,005 | 0,0000 | < 0.00001 | < 0.00001* | 0,0001 | < 0.00001 | < 0.00001* |
| *TCF7L2* | c.1466C>G | rs77673441 | 0,010 | 0,0044 | 0,2310 | 0,5080 | 0,0032 | 0,0890 | 0,2800 |
| *TCF7L2* | c.1466C>A | rs77673441 | 0,030 | 0,0034 | < 0.00001 | < 0.00001* | 0,0219 | 0,7300 | 0,9266 |
| *TP53BP1* | c.5482C>T | rs780546857 | 0,005 | 0,0000 | < 0.00001 | < 0.00001* | - | NA | NA |
| *TSC2* | c.2161G>A | rs768261185 | 0,005 | 0,0000 | < 0.00001 | < 0.00001* | 0,0029 | < 0.00001 | < 0.00001* |
| *WRN* | c.1717A>G | rs150148567 | 0,005 | 0,0014 | 0,1700 | 0,6790 | 0,0877 | 0,0525 | 0,4490 |
| *WRN* | c.1192A>G | rs532554615 | 0,005 | 0,0000 | < 0.00001 | < 0.00001* | 0,0058 | < 0.00001 | < 0.00001* |
| *WRN* | c.2500C>T | rs3087425 | 0,015 | 0,0026 | 0,0005 | 0,0054* | 1,9674 | 0,6420 | 0,8374 |

a Filter A variant from present study

b Filter B variants from present study

* Statistically significant

NA: Not apply

NR: Not reported.
